# Science mapping of COVID-19 contributions in primary health care by OECD countries: A machine learning approach

**DOI:** 10.1177/20552076251389341

**Published:** 2025-10-27

**Authors:** Muhammet Damar, Benita Hosseini, Andrew David Pinto, Omer Aydin, Umit Cali

**Affiliations:** 1 Fethiye Faculty of Business Administration, Management Information Systems, 52986 Mugla Sitki Kocman University, Mugla, Türkiye; 2 Upstream Lab, MAP, Li Ka Shing Knowledge Institute, 508783 Unity Health Toronto, Toronto, Ontario, Canada; 3 Department of Family and Community Medicine, Faculty of Medicine, 7938 University of Toronto, Toronto, Ontario, Canada; 4 Dalla Lana School of Public Health, 7938 University of Toronto, Toronto, Ontario, Canada; 5 Department of Family and Community Medicine, 518773 St. Michael's Hospital, 508783 Unity Health Toronto, Toronto, Ontario, Canada; 6 Computer Science, Electrical and Electronics Engineering, Faculty of Engineering and Natural Sciences, 52953 Manisa Celal Bayar University, Manisa, Turkiye; 7 Department of Management Sciences and Engineering, 8430 University of Waterloo, Waterloo, Canada; 8 Department of Electric Power Engineering, Faculty of Information Technology and Electrical Engineering, 8018 Norwegian University of Science and Technology, Trondheim, Norway; 9 School of Physics, Engineering and Technology, 8748 York University, York, UK

**Keywords:** Canada, OECD countries, COVID-19, primary health care, scientific productivity, research funding, machine learning

## Abstract

**Purpose:**

Our study comprehensively assesses how Canada and Organisation for Economic Co-operation and Development (OECD) countries have supported researchers, research institutes and their scientific productivity in primary health care (PHC), one of the areas most affected by COVID-19.

**Method:**

We analyzed research contributions among OECD countries and assessed their scientific productivity during COVID-19 using bibliometric methods and machine learning techniques. Our analysis includes co-authorship networks, funding patterns, co-citation analysis, thematic mapping, factor analysis, and topic modeling through latent Dirichlet allocation.

**Results:**

This study analyzes 1061 articles and review papers involving 5765 researchers from OECD countries. PHC systems played a crucial role in the global response to SARS-CoV2 but faced significant challenges. Canada ranks third in PHC research output and forth in COVID-19 research among OECD nations. The findings reveal Canada's strong collaborative ties with countries such as the USA, UK, and Australia. However, disparities in PHC scientific productivity across OECD countries remain, with some nations showing minimal progress.

**Conclusions:**

Our study highlights the importance of academic collaboration in addressing pandemic-related crises. The study recommends enhancing international collaboration, led by countries such as Canada, the USA, and the UK, to strengthen PHC systems during global health crises. It is deemed necessary to include experts and academics from the field of PHC in such structures. It also emphasizes the need for academic journals to improve transparency in funding sources through automated extraction of bibliometric data from platforms such as Web of Science and Scopus, which is crucial for shaping future health and education policies.

## Introduction

The Black Death, the Plague,^1^ then the Spanish Flu,^2,3^ and now the spread of the new coronavirus disease (COVID-19) remind us again of how epidemics affect the social order. Since the World Health Organization (WHO) declared the disease a pandemic, countries around the world have adopted measures at various levels to limit the spread of the virus.^4^ The COVID-19 pandemic has had a tremendous impact on health systems in all countries. The rapid progression of the disease has posed a real challenge for the whole world, and health workers and governments have had a significant fight against the pandemic, as the capacity of the health service provided to citizens has been exceeded.^5–7^

The global crisis marked by the deepest public health emergency of the century and the most significant economic downturn since World War II has significantly impeded progress towards achieving the Sustainable Development Goals (SDGs), especially Good Health and Wellbeing (SDG 3) and Reducing Inequality within and Among Countries (SDG 10).^8^ Particularly, governments worldwide, especially in Organisation for Economic Co-operation and Development (OECD) countries, have been compelled to exert comprehensive efforts to restore economic stability and sustain daily life through stimulus packages.^9^ The pandemic has led to a sharp tightening of global financial conditions during the acute phase of the crisis and has resulted in significant economic losses worldwide, potentially with lasting effects.^6,7^ Throughout the COVID-19 pandemic, policymakers aim to increase consumption and investment by providing stimulus packages. These stimulus packages encompass various fiscal support measures with different public financial implications during the COVID-19 pandemic.^10^ Similarly, as an OECD country, the Canadian government has implemented similar economic and fiscal measures. Our study comprehensively assesses how Canada and OECD countries have supported researchers, research institutes and their scientific productivity in primary health care (PHC), the field most affected by COVID-19. For this purpose, bibliometric methods and machine learning techniques are utilized.

## Background

There are numerous studies in the literature that focus on the problems experienced by OECD countries during the COVID-19 pandemic. Rathnayaka et al.^9^ examined the determinants of financial support during the COVID-19 pandemic in 34 leading OECD countries representing the OECD. They focused on whether the decisions were in line with SDG 3 and SDG 10.^9^ Wildman^11^ conducted research to determine the relationship between income inequality and COVID-19 cases and deaths in OECD countries. Palmer and Smal^12^ conducted a review of government policies in four OECD countries and how they would impact young people and young adults in these countries. Additionally, in the COVID-19 and OECD countries search conducted based on article titles on the Web of Science (WoS), publications were found on various topics such as the state of domestic tourism post-COVID-19,^13^ the dimensions of the COVID-19 pandemic for OECD countries,^14^ income inequality and COVID-19 mortality rates for OECD countries,^15^ the effects of the COVID-19 outbreak on the energy and economic sectors,^16^ assessment of adherence to the Mediterranean diet and COVID-19 cases for 24 OECD countries,^17^ evaluation of government “blame” and “credit” communication activities through tweets for four OECD countries,^18^ the impact of COVID-19 on economic growth,^19^ progress in the green finance sector with a focus on OECD countries,^20^ and the relationship between organ donation rates and COVID-19 vaccination status.^21^ However, no publication questioning the funding status of articles related to the COVID-19 topic produced by OECD countries during the COVID-19 process has been accessed in the literature. As known, support for scientific research during such crises is of critical importance.

Bibliometrics is a fundamental tool for monitoring scientific productivity and progress in a given field, and in health, bibliometrics is often used to measure the impact of research articles.^22^ When the literature was examined, studies examining the effect of COVID-19 to many different fields were found. A search with words related to COVID-19 through research titles showed that 574 bibliometric studies were conducted in 77 different research fields. Bibliometric studies have been found in many different research areas such as business and economics,^23^ bioinformatics,^24^ immunology,^25^ nursing,^26^ health care sciences services,^27^ urology,^28^ cardiac cardiovascular systems,^29^ endocrinology metabolism.^30^ Also, bibliometric studies and latent Dirichlet allocation (LDA) analyses have been realized in different periods in the field of PHC and to analyze different aspects of COVID-19 research. In the related field, global scientific research on sars-cov-2 vaccines,^31^ physical activity and COVID-19,^32^ nanotechnology and COVID-19,^33^ analyses of PHC journals,^34^ LDA topic modeling for nursing research,^35^ topic modeling-based analysis of diabetes,^36^ rheumatology and COVID-19 researches,^37^ telemedicine in COVID-19,^38^ COVID-19 and urology^28^ have been studied. In addition, many studies have focused on the development of PHC general literature in different countries such as Africa,^39^ Latin America,^40^ and India.^41^

When bibliometric studies related to COVID-19 and the field of PHC were evaluated, two studies were found in the literature.^34,41^ The first study is a local investigation examining the trend of COVID-19 publications in India specifically in the PHC literature,^41^ while the second study is a bibliometric analysis of publications in the Journal of Family Medicine and PHC over a five-year period.^34^ No research was found in the literature that specifically examines COVID-19 research in the field of PHC. Additionally, no study was found in the literature that analyzes the funding status of COVID-19 research produced in Canada or OECD countries using bibliometric methods and LDA topic analysis machine learning methods. This study provides important reference information for the PHC literature focusing on OECD countries and Canada by comprehensively evaluating high-quality articles in the field, aiming to offer insights for potential future pandemic scenarios.

## Materials and methods

### Objectives of the study and research questions

As is well known, bibliometric studies enable us to analyze the scientific productivity of countries or institutions on a specific topic or field through bibliometric datasets. Scientific productivity refers to the concept of scientific output and denotes the scientific contributions produced by researchers within a specific timeframe. These outputs may include articles, books, conference presentations, patents, or other scientific works. In our study, scientific productivity specifically refers to the scientific output of researchers from OECD countries, as measured through research and review articles published in prominent journals indexed by WoS in the field of PHC research. Additionally, using the most popular topic modeling tool, LDA analysis, we can discover latent themes within a collection of documents, classify the general contents of documents, and determine the topics to which a document belongs based on the words within it.^42,43^ Our research aims to address the following questions based on the article data obtained for Canada and OECD countries:

- Key contributions to COVID-19 research by Canadian and OECD Countries’ researchers?
- Key contributions to COVID-19 research by Canadian and OECD Countries’ researchers to the PHC research area?
- What impact has it had on primary health care in Canadian and OECD countries’ researchers?
- What do bibliometric studies tell us and what examples are there of coronavirus for Canada and OECD countries?
- How did Canada's and OECD countries’ research output compare to the investments made?
- What differences exist in the support provided by OECD countries and Canada in PHC research on COVID-19?
- How have researchers, affiliations, and country collaborations been realized during the COVID-19 time?
- What are the journals in which the research is published, the distribution of references they use and their citation status?
- What are the most intensely related topics and special topics covered in the studies?
- What is the LDA analysis result for Canadian and OECD Countries’ research's?

### OECD countries

The OECD, established in 1961 based on the Paris Convention signed on 14 December 1960, comprises industrialized and developing countries. It consists of 38 member countries spread across the globe from North and South America to Europe and the Asia-Pacific region (List: Australia, Austria, Belgium, Canada, Chile, Colombia, Costa Rica, The Czech Republic, Denmark, Estonia, Finland, France, Germany, Greece, Hungary, Iceland, Ireland, Israel, Italy, Japan, Korea, Latvia, Lithuania, Luxembourg, Mexico, The Netherlands, New Zealand, Norway, Poland, Portugal, The Slovak Republic, Slovenia, Spain, Sweden, Switzerland, Turkey, The United Kingdom (England, Scotland, Wales, Northern Ireland), The United States of America).^44^ In our study, particular attention was paid to the unique situation of the United Kingdom and Turkey in the WoS list, and our analyses were conducted accordingly.

### Research strings and study design

**Figure 1.**
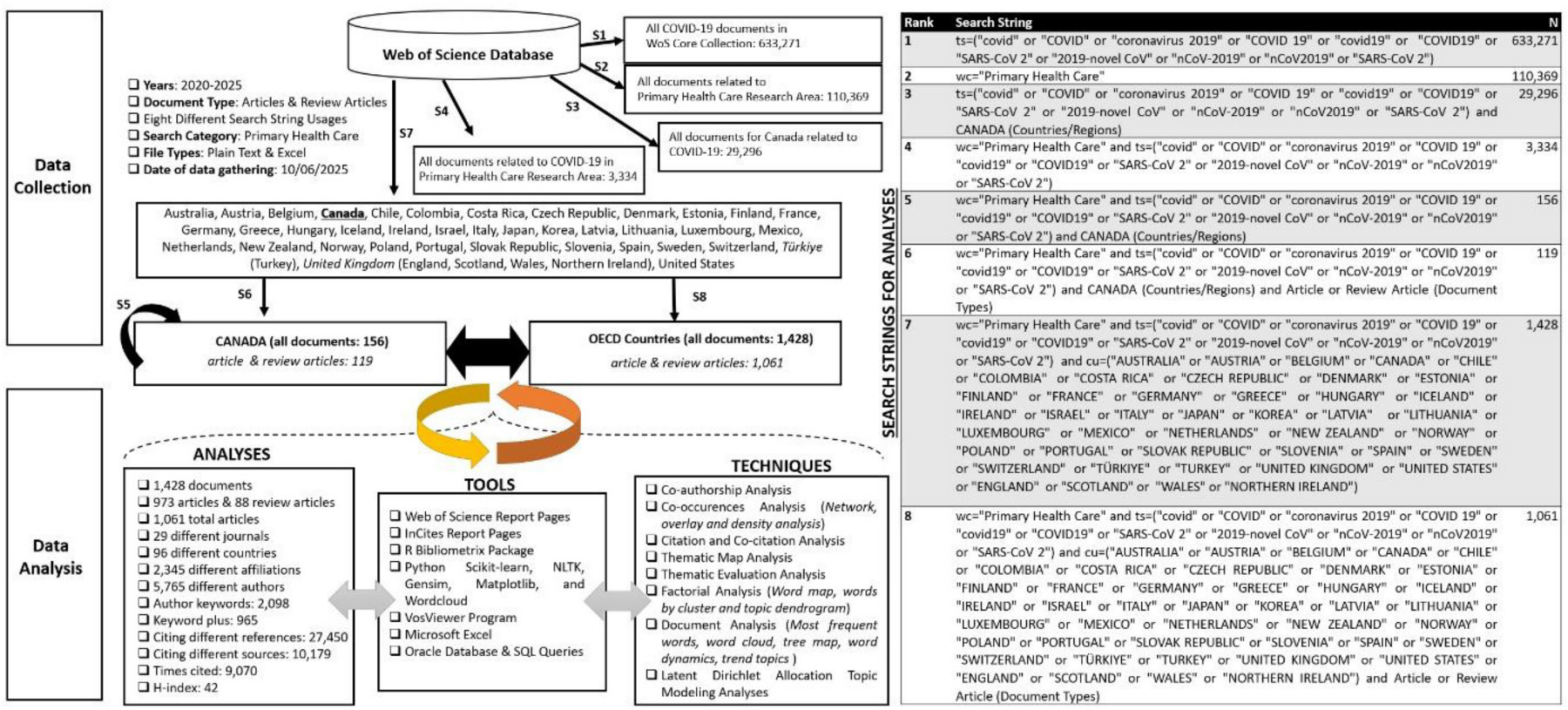
Research methodology and study descriptions.

We retrieved data from the WoS Core Collection on 10 June 2025. The main reason for choosing WoS Core Collection instead of Scopus bibliometric data source is that our research focuses on PHC. PHC is not categorized on Scopus. However, the relevant category is available on WoS.^45^ For data analysis, we used the R Bibliometrix library for Biblioshiny program, and VoSViewer program, and Python programming language with Scikit-Learn,^46^ NLTK,^47^ Gensim,^48^ Matplotlib,^49^ and Wordcloud^50^ libraries. Additionally, Microsoft Excel and Structured Query Language (SQL) were employed for data preprocessing and cleaning. The study design and all techniques used are outlined in Figure 1. The dataset comprises research and review articles, filtered to include only those published between 2020 and 2025. The analysis was conducted on data extracted from the WoS bibliometric database, provided in Plain Text and Excel formats. In addition to the selected tools and techniques employed, the following figure illustrates the search terms utilized to retrieve the relevant dataset from the WoS database. This study provides a detailed comparison of the funding status, research productivity of researchers, institutions, and countries, the most cited works, citation trends, publication journals, and the indexing status of these journals in Canada—an OECD member country—with those of all other OECD nations.

### Why topics analyses, why latent Dirichlet allocation (LDA) and LDA implementation

Topic modeling is an essential technique in text mining and natural language processing that represents words, documents, and corpora as mixture of topics, where a topic is defined as a distribution of words. In this approach, each document contains its own proportion of topics based on the words it contains.^42,51^ Topic modeling is a machine learning method used to automatically identify hidden structures in large amounts of text data, that is, the underlying themes or topics that texts contain. This technique enables better classification, analysis and understanding of texts. There are various topic modeling methods^52^ such as Latent Semantic Indexing (LSI),^53^ Probabilistic Latent Semantic Analysis (PLSA),^54^ LDA,^42^ Correlation Explanation (CorEx),^55^ Hierarchical Dirichlet Process (HDP),^56^ Dynamic Topic Modeling (DTM),^57^ BERTopic,^58^ Probabilistic Latent Semantic Analysis (PLSA),^54^ and Neural Topic Models (NTM).^59^ Each method has advantages and disadvantages, and the appropriate method varies depending on the goals of the project, the size of the data set, and the type of content. For instance, LSA offers simplicity and computational efficiency, particularly when applied to smaller datasets.^53^ HDP is a derivative of LDA, but does not need a predetermined number of topics.^56^ BERTopic identifies topics using transformer-based deep learning models.^58^ For our paper, LDA was the appropriate method.

LDA Topic Modeling is an unsupervised learning method because it does not require labeled data to discover hidden structures (topics) in the data. This model attempts to understand the topics between documents by representing each document and word with their probabilities of belonging to specific topics, thereby learning the hidden structures in the data. This process is classified as a machine learning technique because the learning algorithm is based on finding patterns. Li and Lei^43^ examined topic modeling paper between 2000 and 2017. As a result of this analysis, it was determined that LDA is the most popular algorithm in social networks and text analysis topic modeling. Using LDA, the topics in documents can be determined, providing a clear representation of their content.^42^ LDA aims to identify topics that align with the content of each document by modeling both the topic distributions of documents and the word distributions of each topic. This approach helps us to more accurately understand the similarities and differences between documents. LDA is a probabilistic model, meaning it expresses the origin of each word in a document as a probability. This feature enhances the model's flexibility and allows it to better learn the relationships between words within a document. Based on the co-occurrence probabilities of words in documents, LDA assigns each document to multiple topics, acknowledging that a document may not belong to a single topic but instead encompass multiple themes. This approach enables a deeper understanding of the nuances between topics.^42,43,45,51,60^ For instance, consider the topic of “fishing,” which includes words such as “bass,” “anchovy,” “fisherman,” and “fishing boat,” thereby generating meaningful themes. Such themes provide users with more insightful and useful content recommendations or analyses. Additionally, LDA performs efficiently on large datasets, making it a suitable choice for analyzing extensive collections of documents. In our research, analyses are conducted based on various metrics, such as the abstracts, keywords, and titles of numerous articles. In conclusion, LDA is a robust tool for discovering and analyzing latent structures within documents. It is therefore considered a valuable method for topic modeling. For these reasons, LDA has been selected for our research.

In our study, we applied LDA to perform topic modeling on abstracts and titles of research articles related to Canada and other OECD countries. This analysis was carried out using Python with Scikit-learn, NLTK, Gensim, Matplotlib, and Wordcloud libraries. We preprocessed the dataset to prepare it for topic modeling using LDA. The preprocessing steps, performed with Python, included converting text to lowercase, removing punctuation marks and numbers, and eliminating irrelevant words. The text was tokenized using the NLTK library, and lemmatization was performed to merge words with similar meanings. Additionally, stop words were removed.

After preprocessing, we split the dataset into training and validation subsets using the Scikit-learn library. There are two primary hyperparameters α and β that are widely used with the LDA algorithm. α is a hyperparameter that controls the frequency of a document-specific topic in the document. A higher alpha value increases the likelihood that more topics will be found in a document. Conversely, a lower alpha value increases the likelihood that fewer topics will be found in the document. β is a hyperparameter that controls the frequency of the word in a topic. A higher beta value increases the likelihood of more words occurring within a topic. Conversely, a lower beta value increases the likelihood that fewer words will occur within a topic.^61^ At this stage, the text data that went through the pre-processing phase was first trained using machine learning with Python language. Afterwards, the LDA algorithm was applied to the validation data. α and β values were tested with various values, symmetrically and asymmetrically, to determine at which values the model gave successful results. When selecting topics for the LDA analysis, the most suitable word patterns were found to be derived from the six-fold dataset. The perplexity metric value and coherence score have been key metrics in this process. Additionally, it was assessed whether the identified word patterns formed meaningful structures from the perspective of researchers. For this purpose, an evaluation was conducted based on the general titles and abstracts of the articles. Finally, we applied the LDA model to conduct the topic modeling, with visualizations generated using Matplotlib and Wordcloud libraries.

## Results

### General results

A search using the designated keywords related to the coronavirus revealed that a total of 633,271 documents were produced globally. Of these, 29,296 documents were attributed to researchers affiliated with Canada. In the field of PHC research, a total of 110,369 documents were produced, with 3334 documents specifically addressing the relevant topic. Among these, 1428 (including 1061 research articles and review articles) were produced by OECD countries. Canada's contribution within the OECD accounted for 156 documents, including 119 articles (106 research articles and 13 review articles). The annual productivity of these articles was as follows: 2025 (10 articles, 8.40%), 2024 (23 articles, 19.32%), 2023 (22 articles, 18.48%), 2022 (31 articles, 26.05%), 2021 (26 articles, 21.84%), and 2020 (7 articles, 5.88%). Among the 1061 articles produced by OECD-affiliated researchers, 673 (63.43%) were published in journals indexed in the Science Citation Index Expanded (SCI-Expanded). In comparison, 94 articles (78.99%) from Canadian researchers were published in SCI-Expanded journals. Additionally, 388 OECD-affiliated articles (36.56%) appeared in journals indexed in the Emerging Sources Citation Index (ESCI), while 25 Canadian articles (21.00%) were published in ESCI-indexed journals. Similarly, 83 OECD articles (7.82%) were published in the Social Sciences Citation Index (SSCI), with 7 Canadian articles (5.88%) appearing in SSCI-indexed journals.

### Authors, affiliations, and countries analyses

**Figure 2.**
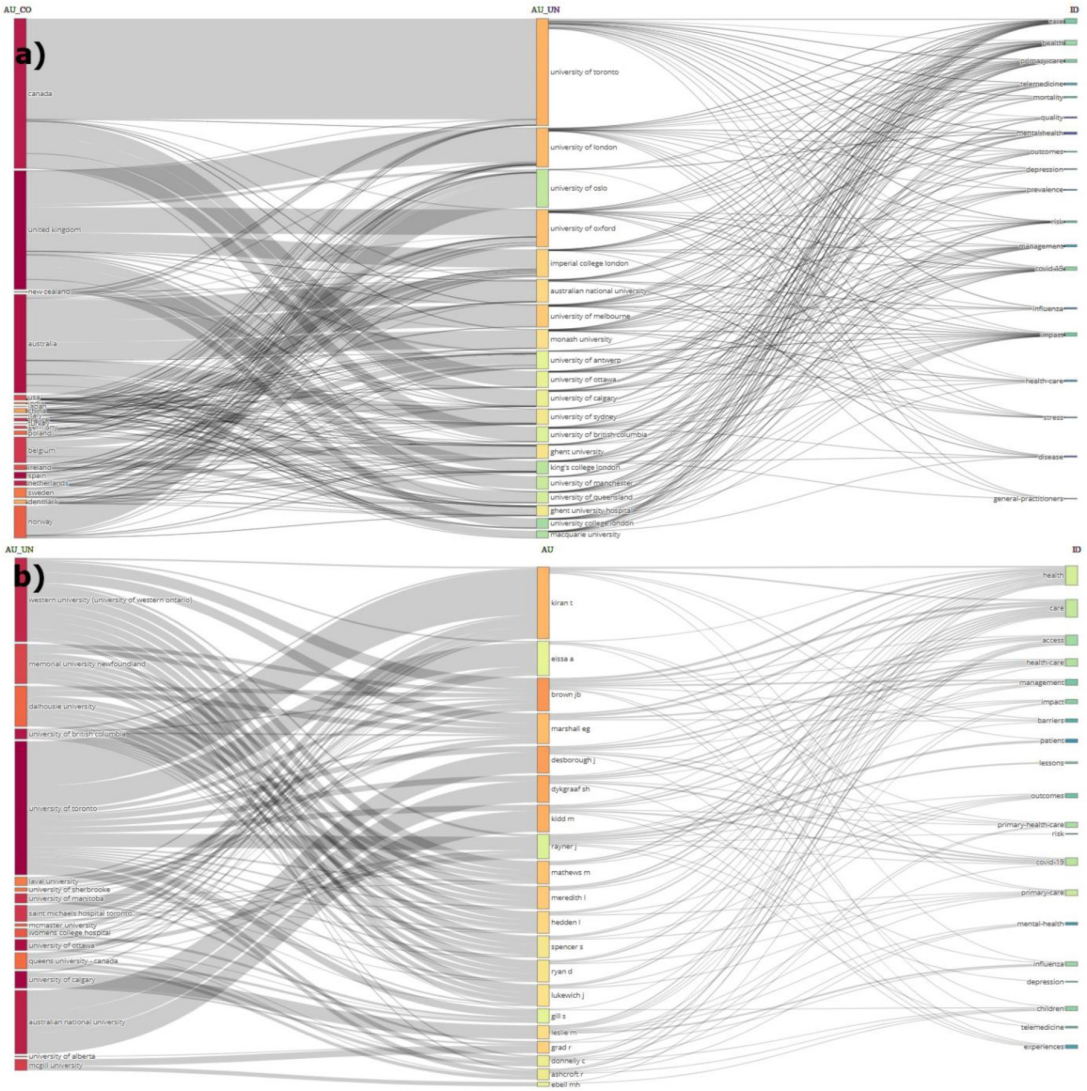
Three-field plot analyses with author country, affiliations, and keywords (a) OECD countries, and (b) Canada.

The focused research topics by OECD countries, institutions, and researchers are presented in Figure 2(a), while Figure 2(b) shows the research topics addressed by Canadian institutions and researchers.

**Figure 3.**
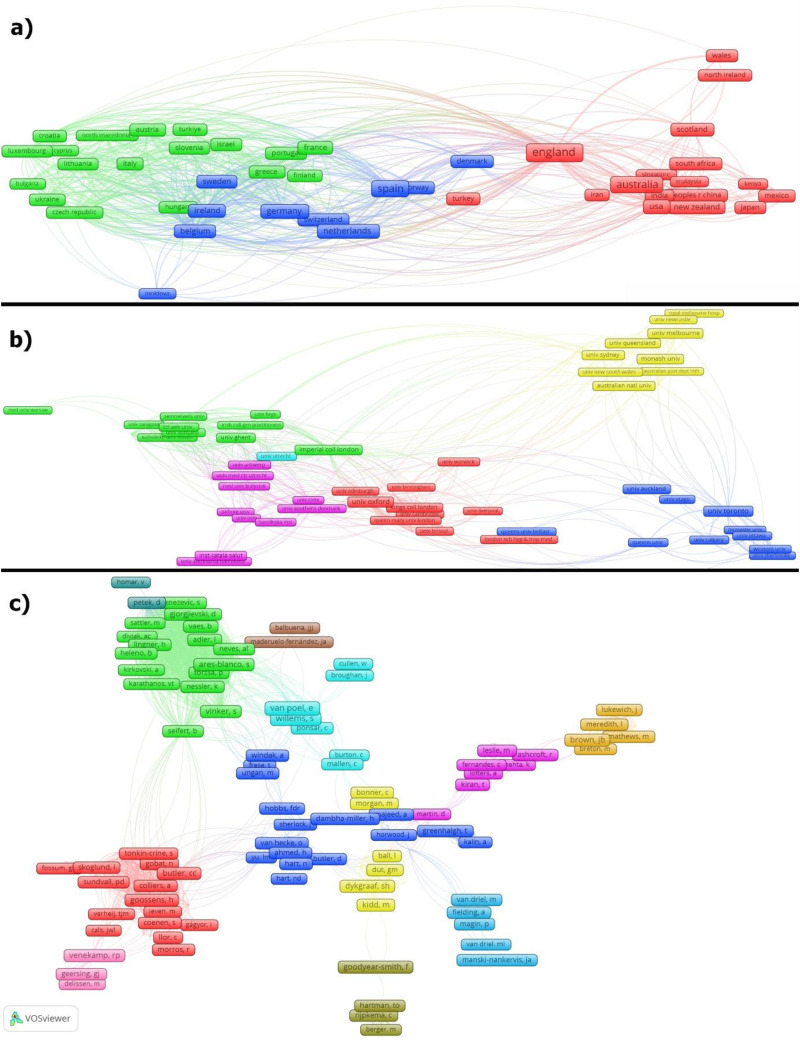
Co-authorship network analyses for OECD countries (a) countries, (b) Institutions, (c) authors.

The articles related to the coronavirus in PHC research from OECD countries involved 2345 different institutions and 5765 researchers from 96 countries. Whereas, the 119 articles produced by Canada involved 286 different institutions and 692 researchers from 33 countries. Publication statistics for OECD countries are provided in Table 1, publication statistics for OECD countries in Table 2, and the list of researchers in OECD countries in Appendix 1. Additionally, publication statistics for Canadian institutions are presented in Table 3, and the list of Canadian researchers is provided in Appendix 2. Moreover, the list of collaborating countries and the collaboration network for OECD countries are detailed in Appendix 3, while Appendix 4 contains the corresponding information for Canada. In addition to these analyses, co-authorship network analyses for OECD countries have been analyzed by country, institution, and researcher and shared on Figure 3.

**Table 1.** Scientific productivity of OECD countries on COVID-19 in primary health care research area.

Rank	Countries	HI	ACPA	N	%	Rank	Countries	HI	ACPA	N	%
0	United Kingdom	27	12.06	284	26.76	22	Greece	6	11.40	20	1.88
1	England	26	11.99	248	23.37	23	Switzerland	8	9.80	20	1.88
2	Spain	14	6.17	170	16.02	24	Israel	9	11.58	19	1.79
3	Australia	16	8.12	161	15.17	25	Mexico	4	3.58	19	1.79
4	Canada	13	6.80	119	11.21	28	Austria	5	15.33	18	1.69
5	USA	12	10.52	64	6.03	28	Slovenia	5	7.06	18	1.69
6	Netherlands	10	12.62	61	5.74	30	Portugal	5	8.00	17	1.60
7	Germany	15	12.12	58	5.46	31	North Ireland^a^	6	9.00	14	1.32
8	Belgium	9	8.18	50	4.71	33	Hungary	4	3.92	13	1.22
9	New Zealand	10	9.88	43	4.05	34	South Korea	3	2.54	13	1.22
10	Turkiye^b^	13	10.37	43	4.05	35	Finland	4	4.50	10	0.94
11	France	10	7.46	39	3.67	39	Czech Republic	4	5.00	8	0.75
12	Poland	8	6.86	37	3.48	41	Lithuania	3	3.25	8	0.75
13	Scotland^a^	9	14.31	36	3.39	43	Luxembourg	4	7.29	7	0.66
14	Ireland	8	8.09	35	3.29	57	Colombia	3	55.50	4	0.37
15	Sweden	9	8.10	31	2.92	66	Chile	2	3.00	3	0.28
16	Norway	6	4.50	28	2.63	67	Estonia	2	2.33	3	0.28
17	Denmark	6	5.65	26	2.45	73	Latvia	2	9.50	2	0.18
18	Japan	6	3.40	25	2.35	0	Costa Rica	0	0	0	0
19	Italy	9	8.92	24	2.26	0	Slovak Republic	0	0	0	0
20	Wales^a^	7	8.65	23	2.16	0	Iceland	0	0	0	0

ACPA: average citation per articles; N: document count; HI: H-index; TC: times cited; N: article count.

a United Kingdom: England, Scotland, Wales, Northern Ireland.

b Turkiye: Turkey and Turkiye.

**Table 2.** Scientific productivity of OECD countries’ institutions on COVID-19 in primary health care research area.

Rank	Affiliations	Country	TC	HI	ACPA	N	% 1.061
1	University of London	England	721	16	10.15	71	6.69
2	University of Oxford	England	999	16	17.22	58	5.46
3	University of Toronto	Canada	559	12	10.55	53	4.99
4	University of Melbourne	Australia	301	7	8.60	35	3.29
5	Imperial College London	England	484	9	14.24	34	3.20
6	Monash University	Australia	349	8	10.58	33	3.11
7	Ghent University	Belgium	105	5	3.89	27	2.54
8	Ghent University Hospital	Belgium	99	4	3.81	26	2.45
9	King S College London	England	348	10	13.92	25	2.35
10	University of Sydney	Australia	144	5	5.76	25	2.35
11	Institut Català de la Salut	Spain	413	7	17.21	24	2.26
12	University of Queensland	Australia	159	7	6.63	24	2.26
13	University College London	England	191	7	8.68	22	2.07
14	University of Otago	New Zealand	272	6	12.36	22	2.07
15	Australian National University	Australia	423	7	20.14	21	1.97
16	Macquarie University	Australia	62	5	3.10	20	1.88
17	Queen Mary University London	England	120	7	6.00	20	1.88
18	University of Auckland	New Zealand	162	7	8.10	20	1.88
19	Utrecht University	Netherlands	145	5	7.25	20	1.88
20	Utrecht University Medical Center	Netherlands	142	5	7.47	19	1.79

ACPA: average citation per articles; N: document count; HI: H-index; TC: times cited; N: article count.

**Table 3.** Canadian researchers’ institutional scientific productivity on COVID-19 in primary health care research field.

Rank	Affiliations	Country	TC	HI	ACPA	N	%
1	University of Toronto	Canada	559	12	10.55	53	44.53
2	Western University	Canada	122	7	7.18	17	14.28
3	University of Calgary	Canada	56	4	3.50	16	13.44
4	University of British Columbia	Canada	77	5	5.13	15	12.60
5	University of Ottawa	Canada	105	5	7.50	14	11.76
6	Mcgill University	Canada	41	3	3.42	12	10.08
7	Dalhousie University	Canada	45	4	4.09	11	9.24
8	Saint Michaels Hospital Toronto	Canada	90	5	8.18	11	9.24
9	Queens University Canada	Canada	142	3	14.20	10	8.40
10	Mcmaster University	Canada	37	3	4.11	9	7.56
11	Memorial University Newfoundland	Canada	46	4	5.11	9	7.56
12	University of Manitoba	Canada	41	4	5.13	8	6.72
13	Australian National University	Australia	135	4	19.29	7	5.88
14	Flinders University South Australia	Australia	136	4	19.43	7	5.88
15	University of Alberta	Canada	27	3	3.86	7	5.88
16	University of Sherbrooke	Canada	148	5	21.14	7	5.88
17	Womens College Hospital	Canada	35	3	5.00	7	5.88
18	Alberta Health Services	Canada	10	2	1.67	6	5.04
19	Laval University	Canada	35	2	5.83	6	5.04
20	Li Ka Shing Knowledge Institute	Canada	47	4	7.83	6	5.04

ACPA: average citation per articles; N: document count; HI: H-index; TC: times cited; N: article count.

### Document, citation, and references analyses

**Figure 4.**
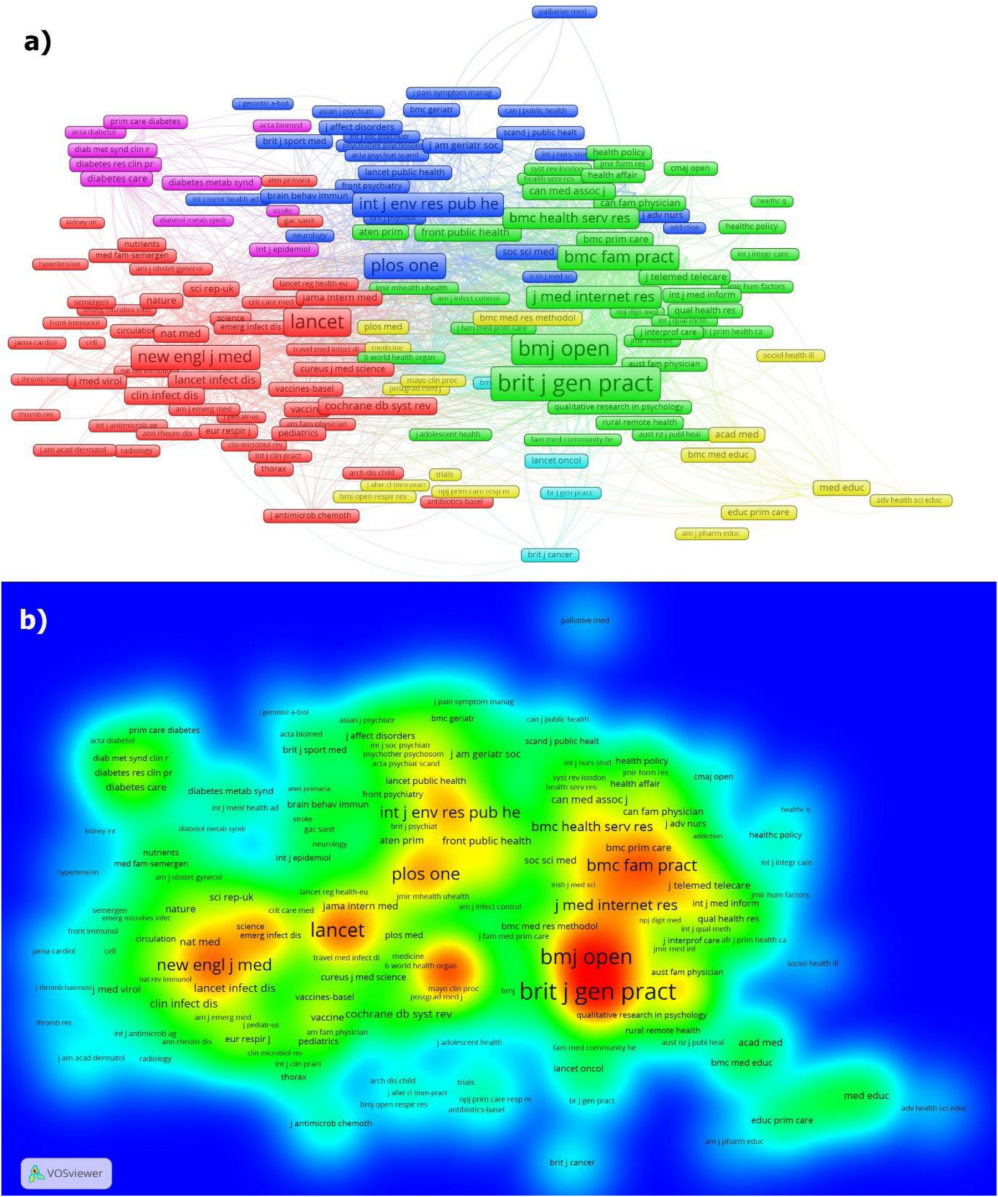
OECD countries’ co-citation network (a) and density (b) analyses for sources.

Publications from OECD countries addressing COVID-19 appeared in 29 different journals, with a total of 10,179 unique sources and 27,450 references. The Canadian 119 articles were published in 19 different journals, using 1964 unique sources and 3852 references. The journals with the highest number of publications on COVID-19 from OECD countries are provided in Table 4, while the most cited publications from OECD countries are listed in Table 5. Similarly, the journals with the highest number of publications on COVID-19 from Canada are presented in Table 6, and the most cited publications from Canada are listed in Table 7. In addition to these analyses, the co-citation network and density analysis for OECD countries is given in Figure 4.

**Table 4.** Journals with the most intensive publications on COVID-19 in OECD countries in primary health care research area.

Rank	Journal	JIF	Research domain	SCIE/ SSCI/ ESCI	ACPA	HI	N	%
1	BMC Primary Care	2.0	Primary Health Care; Medicine, General & Internal	SCIE	4.01	12	143	13.47
2	BJGP Open	2.5	General & Internal Medicine	ESCI	6.98	13	93	8.76
3	British Journal of General Practice	5.3	Primary Health Care; Medicine, General & Internal	SCIE	23.01	20	73	6.88
4	Medicina De Familia Semergen	0.9	Primary Health Care	ESCI	3.16	8	70	6.59
5	Family Practice	2.4	Primary Health Care; Medicine, General & Internal	SCIE	13.14	12	59	5.56
6	Atencion Primaria	1.8	Primary Health Care; Medicine, General & Internal	SCIE	8.76	10	54	5.09
7	Journal of Primary Care and Community Health	3.0	Primary Health Care	ESCI	15.89	13	54	5.09
8	Australian Journal of General Practice	1.6	Primary Health Care; Medicine, General & Internal	SCIE	7.25	11	52	4.90
9	European Journal of General Practice	2.3	Primary Health Care; Medicine, General & Internal	SCIE	11.53	9	43	4.05
10	Australian Journal of Primary Health	1.2	Health Care Sciences & Services; Health Policy & Services; Primary Health Care; Public, Environmental & Occupational Health	SCIE & SSCI	4.17	6	42	3.95
11	Journal of Family Medicine and Primary Care	1.1	Primary Health Care	ESCI	2.88	6	41	3.86
12	Education for Primary Care	1.5	Primary Health Care	ESCI	6.00	8	35	3.29
13	Journal of Primary Health Care	1.1	Primary Health Care	ESCI	3.79	6	33	3.11
14	Primary Care Diabetes	2.6	Endocrinology & Metabolism; Primary Health Care	SCIE	9.53	10	32	3.01
15	BMC Family Practice	3.2	Primary Health Care; Medicine, General & Internal	SCIE	33.81	17	31	2.92
16	Family Medicine and Primary Care Review	0.5	Primary Health Care	ESCI	1.00	3	27	2.54
17	Primary Health Care Research and Development	1.6	Primary Health Care	SCIE	6.00	7	25	2.35
18	Annals of Family Medicine	4.4	Primary Health Care; Medicine, General & Internal	SCIE	8.61	7	23	2.16
19	Scandinavian Journal of Primary Health Care	1.9	Health Care Sciences & Services; Primary Health Care; Medicine, General & Internal	ESCI	2.64	3	22	2.07
20	Canadian Family Physician	2.4	Primary Health Care; Medicine, General & Internal	SCIE	2.95	5	21	1.97

N: document count; JIF: journal impact factor for 2023 years; HI: H-index; ACPA: average citation per articles; N: article count.

**Table 5.** OECD countries’ most cited publications on COVID-19 in primary health care research area.

Rank	Title	Journal	JIF	Authors	Year	C
1	Implementation of remote consulting in UK primary care following the COVID-19 pandemic: a mixed-methods longitudinal study	British Journal of General Practice	5.3	Murphy, M; Scott, LJ; (…); Horwood, J	2021	280
2	Impact of COVID-19 on loneliness, mental health, and health service utilisation: a prospective cohort study of older adults with multimorbidity in primary care	British Journal of General Practice	5.3	Wong, SYS; Zhang, DX; (…); Mercer, SW	2020	250
3	Post-acute and long-COVID-19 symptoms in patients with mild diseases: a systematic review	Family Practice	2.4	van Kessel, SAM; Hartman, TCO; (…); van Jaarsveld, CHM	2022	225
4	Lessons on the COVID-19 pandemic, for and by primary care professionals worldwide	European Journal of General Practice	2.3	Rawaf, S; Allen, LN; (…); van Weel, C	2020	212
5	Implementation and Usefulness of Telemedicine During the COVID-19 Pandemic: A Scoping Review	Journal of Primary Care And Community Health	3.0	Hincapié, MA; Gallego, JC; (…); Escobar, MF	2020	207
6	Telemedicine in the face of the COVID-19 pandemic	Atencion Primaria	1.8	Vidal-Alaball, J; Acosta-Roja, R; (…); Seguí, FL	2020	204
7	Telehealth consultations in general practice during a pandemic lockdown: survey and interviews on patient experiences and preferences	BMC Family Practice	3.2	Imlach, F; McKinlay, E; (…); McBride-Henry, K	2020	186
8	The impact of COVID-19 on chronic care according to providers: a qualitative study among primary care practices in Belgium	BMC Family Practice	3.2	Danhieux, K; Buffel, V; (…); van Olmen, J	2020	126
9	The effectiveness of teleconsultations in primary care: systematic review	Family Practice	2.4	de Albornoz, SC; Sia, KL and Harris, A	2020	121
10	Mental Health Burden of the COVID-19 Outbreak in Germany: Predictors of Mental Health Impairment	Journal of Primary Care And Community Health	3.0	Bäuerle, A; Steinbach, J; (…); Skoda, EM	2020	107
11	Impact of COVID-19 on migrants’ access to primary care and implications for vaccine roll-out: a national qualitative study	British Journal of General Practice	5.3	Knights, F; Carter, J; (…); Hargreaves, S	2021	105
12	Telehealth challenges during COVID-19 as reported by primary healthcare physicians in Quebec and Massachusetts	BMC Family Practice	3.2	Breton, M; Sullivan, EE; (…); McAlearney, AS	2021	97
13	Reorganisation of primary care for older adults during COVID-19: a cross-sectional database study in the UK	British Journal of General Practice	5.3	Joy, M; McGagh, D; (…); de Lusignan, S	2020	94
14	A Multidisciplinary NHS COVID-19 Service to Manage Post-COVID-19 Syndrome in the Community	Journal of Primary Care And Community Health	3.0	Parkin, A; Davison, J; (…); Sivan, M	2021	91
15	Primary care in the time of COVID-19: monitoring the effect of the pandemic and the lockdown measures on 34 quality of care indicators calculated for 288 primary care practices covering about 6 million people in Catalonia	BMC Family Practice	3.2	Coma, E; Mora, N; (…); Medina, M	2020	91

JIF: journal impact factor for 2023 years; C: citation.

**Table 6.** Journals in primary health care research area where Canadian researchers publish most intensively on COVID-19.

Rank	Journal	JIF	Research domain	SCIE/ SSCI/ ESCI	ACPA	HI	N	%
1	BMC Primary Care	2.0	Primary Health Care; Medicine, General & Internal	SCIE	3.00	7	34	28.57
2	Canadian Family Physician	2.4	Primary Health Care; Medicine, General & Internal	SCIE	2.95	5	21	17.64
3	Journal of Primary Care and Community Health	3.0	Primary Health Care	ESCI	4.25	4	12	10.08
4	Annals of Family Medicine	4.4	Primary Health Care; Medicine, General & Internal	SCIE	8.18	5	11	9.24
5	Family Practice	2.4	Primary Health Care; Medicine, General & Internal	SCIE	17.22	5	9	7.56
6	American Family Physician	3.8	Primary Health Care; Medicine, General & Internal	SCIE	1.00	1	4	3.36
7	Australian Journal of General Practice	1.6	Primary Health Care; Medicine, General & Internal	SCIE	9.25	3	4	3.36
8	Journal of Family Medicine and Primary Care	1.1	Primary Health Care	ESCI	2.00	2	4	3.36
9	BMC Family Practice	3.2	Primary Health Care; Medicine, General & Internal	SCIE	68.00	3	3	2.52
10	Family Medicine and Community Health	2.6	Primary Health Care	ESCI	4.33	2	3	2.52
11	Journal of The American Board of Family Medicine	2.4	Primary Health Care; Medicine, General & Internal	SCIE	6.67	2	3	2.52
12	BJGP Open	2.5	General & Internal Medicine	ESCI	3.50	0	2	1.68
13	Education for Primary Care	1.5	Primary Health Care	ESCI	7.5	2	2	1.68
14	Physician and Sportsmedicine	1.9	Primary Health Care; Orthopedics; Sport Sciences	SCIE	12.50	1	2	1.68
15	African Journal of Primary Health Care Family Medicine	1.2	Primary Health Care	ESCI	2.00	1	1	0.84
16	Australian Journal of Primary Health	1.2	Health Care Sciences & Services; Health Policy & Services; Primary Health Care; Public, Environmental & Occupational Health	SCIE & SSCI	1.00	1	1	0.84
17	British Journal of General Practice	5.3	Primary Health Care; Medicine, General & Internal	SCIE	11.00	1	1	0.84
18	European Journal of General Practice	2.3	Primary Health Care; Medicine, General & Internal	SCIE	2.00	1	1	0.84
19	Family Medicine	1.8	Primary Health Care; Medicine, General & Internal	SCIE	0.00	0	1	0.84

N: document count; JIF: journal impact factor for 2023 years; HI: H-index; ACPA: average citation per articles; N: article count.

**Table 7.** Most cited publications on COVID-19 by Canadian researchers in primary health care research area.

Rank	Title	Journal	JIF	Authors	Year	C
1	Telehealth challenges during COVID-19 as reported by primary healthcare physicians in Quebec and Massachusetts	BMC Family Practice	3.2	Breton, M; Sullivan, EE; (…); McAlearney, AS	2021	97
2	Ensuring the continuation of routine primary care during the COVID-19 pandemic: a review of the international literature	Family Practice	2.4	Matenge, S; Sturgiss, E; (…); Kidd, M	2021	57
3	Primary care teams’ experiences of delivering mental health care during the COVID-19 pandemic: a qualitative study	BMC Family Practice	3.2	Ashcroft, R; Donnelly, C; (…); Brown, JB	2021	55
4	Lessons for the global primary care response to COVID-19: a rapid review of evidence from past epidemics	Family Practice	2.4	Desborough, J; Dykgraaf, SH; (…); Kidd, M	2021	54
5	Interprofessional primary care during COVID-19: a survey of the provider perspective	BMC Family Practice	3.2	Donnelly, C; Ashcroft, R; (…); Miller, J	2021	52
6	Family Physicians Stopping Practice During the COVID-19 Pandemic in Ontario, Canada	Annals of Family Medicine	4.4	Kiran, T; Green, ME; (…); Glazier, RH	2022	27
7	Does wearing a mask while exercising amid COVID-19 pandemic affect hemodynamic and hematologic function among healthy individuals? Implications of mask modality, sex, and exercise intensity	Physician and Sportsmedicine	1.9	Ahmadian, M; Ghasemi, M; (…); Roshan, VD	2021	24
8	Double Jeopardy: Maintaining Livelihoods or Preserving Health? The Tough Choices Sex Workers Faced during the COVID-19 Pandemic	Journal of Primary Care and Community Health	3.0	Shareck, M; Hassan, M; (…); O'Campo, P	2021	20
9	The perspective of Canadian health care professionals on abortion service during the COVID-19 pandemic	Family Practice	2.4	Ennis, M; Wahl, K; (…); Norman, W	2021	18
10	In, But Out of Touch: Connecting With Patients During the Virtual Visit	Annals of Family Medicine	4.4	Kelly, MA and Gormley, GJ	2020	17
11	The importance of consistent advice during a pandemic An analysis of Australian advice regarding personal protective equipment in healthcare settings during COVID-19	Australian Journal of General Practice	1.6	Desborough, J; Dykgraaf, SH; (…); Kidd, M	2020	15
12	Best Practices for COVID-19 Mass Vaccination Clinics	Annals of Family Medicine	4.4	Shakory, S; Eissa, A; (…); Pinto, AD	2022	14
13	A simulation training course for family medicine residents in China managing COVID-19	Australian Journal of General Practice	1.6	Shi, DD; Lu, H; (…); Xu, ZQ	2020	14
14	Implementing High-Quality Primary Care Through a Health Equity Lens	Annals of Family Medicine	4.4	Eissa, A; Rowe, R; (…); Rodríguez, JE	2022	13
15	Caring for refugees and newcomers in the post-COVID-19 era	Canadian Family Physician	2.4	Arya, N; Redditt, VJ; (…); Pottie, K	2021	13

JIF: journal impact factor for 2023 years; C: citation.

Based on Bradford's Law,^62,63^ the primary zone journals for OECD researchers were BMC Primary Care, BJGP Open, British Journal of General Practice, and Medicina De Familia-Semergen. Similarly, for analyses of research from Canada, the two prominent primary zone journals are BMC Primary Care and Canadian Family Physician.

### Content analyses and latent Dirichlet allocation

**Figure 5.**
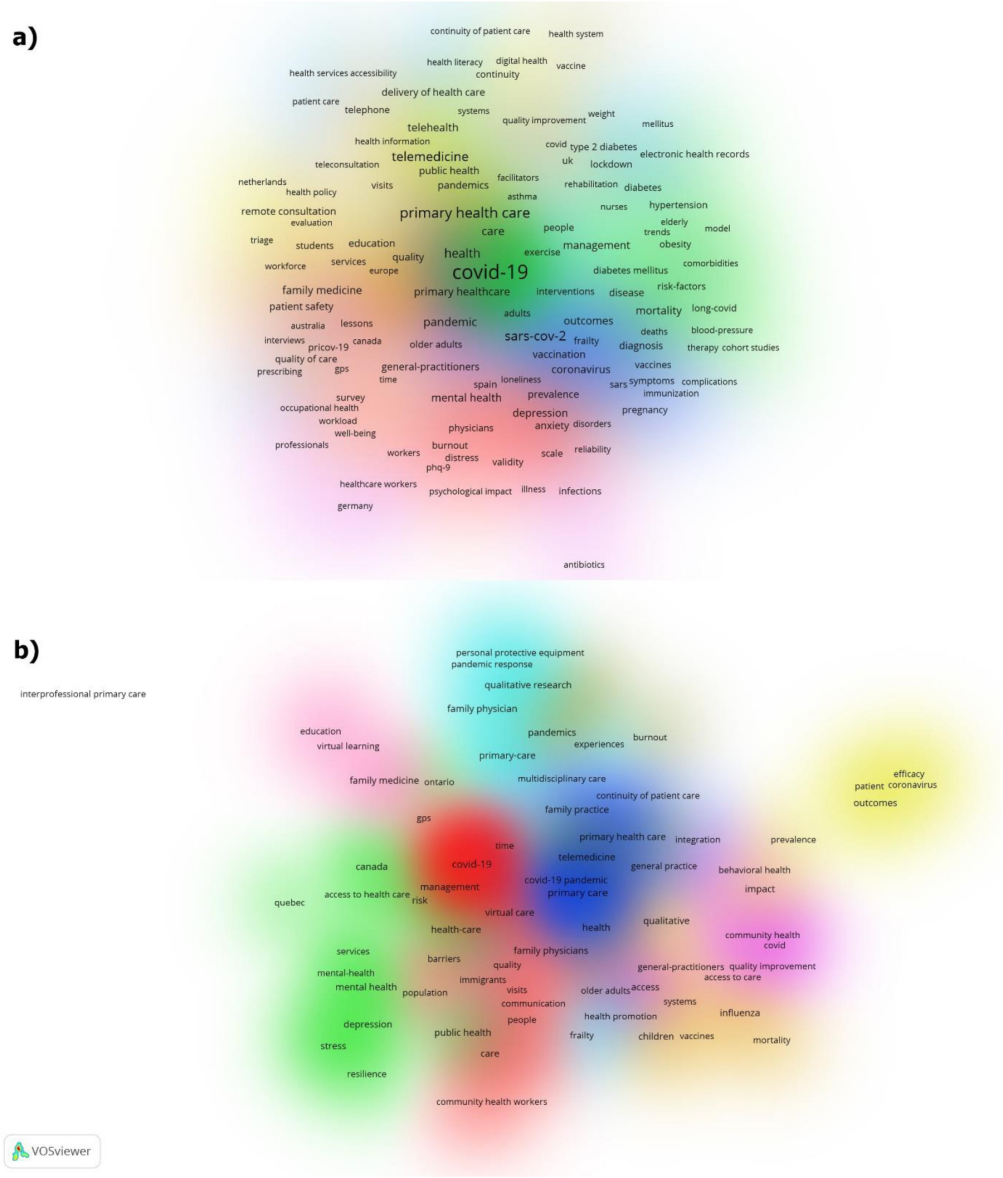
Co-occurrence network analyses keywords (a) OECD countries, and (b) Canada.

Prominent research topics in OECD countries included telemedicine, telehealth, remote consultation, teleconsultation, virtual care, delivery of health care, access to care, health policy, health promotion, health services accessibility, healthcare workers, mental health, depression, stress, anxiety, burnout, workload, workforce, medical education, diabetes, vaccination, chronic disease, multimorbidity, obesity, physical activity, pregnancy, geriatrics, health system, and hospitalization (Figure 5(a)). A similar trend was observed in Canadian research (Figure 5(b)). Keywords such as pandemic, COVID-19, general practice, family medicine, community health, general practitioners, public health, and PHC were most frequently used in research from both Canada and other OECD countries (Figure 5).

**Figure 6.**
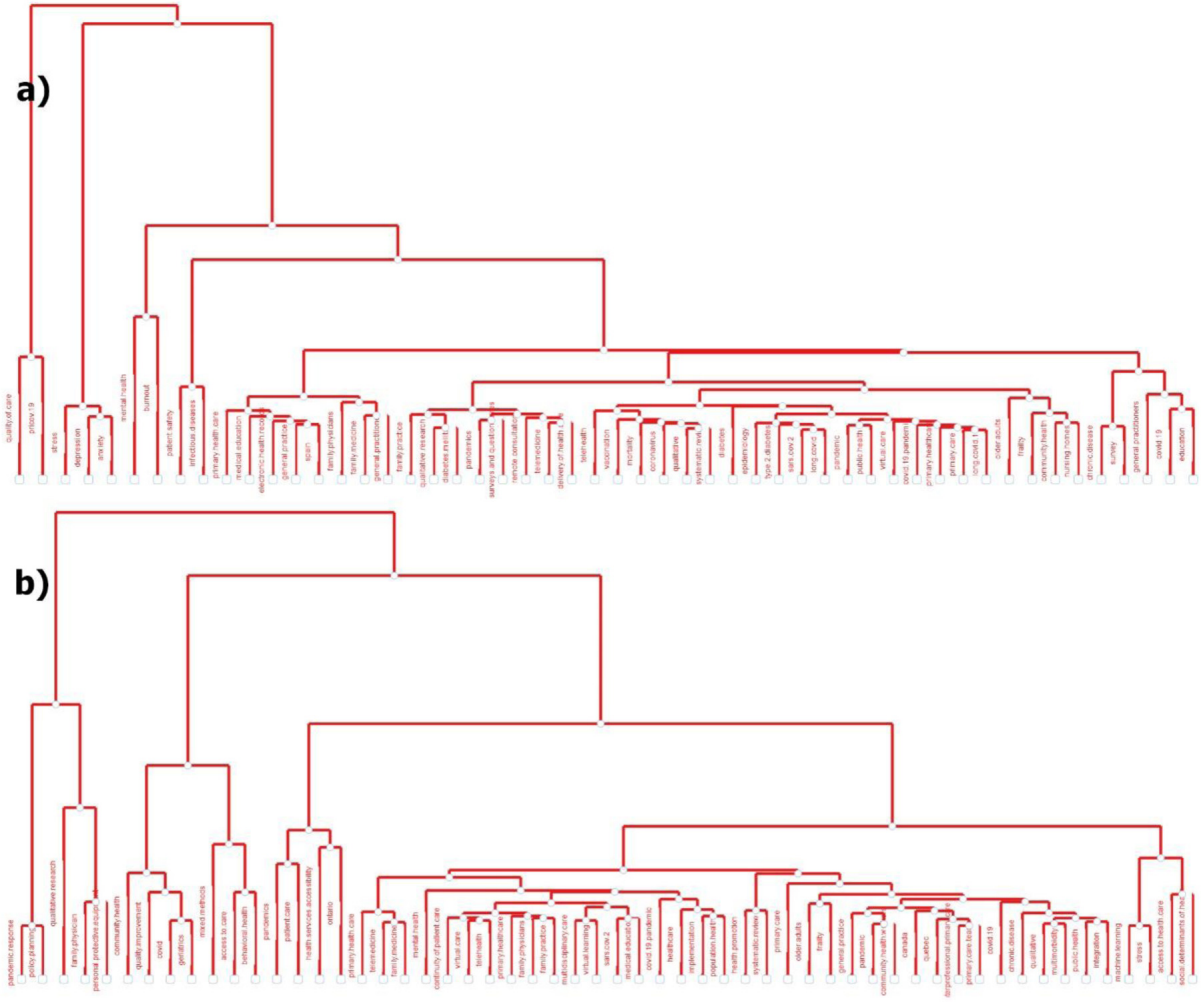
Factorial analysis as a topic dendrograms for article keywords (a) OECD countries, and (b) Canada.

Factor analysis is a technique that reduces large datasets into clusters of independent variables. Using the keywords from OECD (Figure 6(a)) and Canadian (Figure 6(b)) research, we generated topic dendrograms through factor analysis. The prominent research topics in the titles and abstracts of OECD and Canadian publications identified through LDA analyses topic modeling, as presented in Table 8. The analysis was based on article keywords, keywords plus, titles, and abstracts and most appropriate six topics for OECD countries and Canada are represented in Table 8. For OECD countries, the perplexity metric value is 2752.627, while the coherence score is 0.866. For Canada, the perplexity metric value is 3077.655, whereas the coherence score is 0.456.

**Table 8.** Cluster title and description with LDA analyses for article title and abstract.

	Rank	Cluster title	Narrative description of content
OECD	1	Psychological Effects and Burnout in Health Workers	This topic addresses the psychological effects of the pandemic on healthcare workers (especially “resident” groups such as physician assistants), such as emotional exhaustion, burnout, and sleep problems, especially in the post-pandemic period.
	2	Antibiotic Use, Reactions and Migrant Health	This topic covers antibiotic use, side effects (ADRs), access to healthcare services for migrants/unaccompanied minors, and resistance during COVID-19.
	3	Effects of COVID-19 on General Public Health	The public health impacts of COVID-19 include general epidemiological issues such as transmission risk, psychological effects (anxiety, depression), vaccination, and mortality rates.
	4	Health Programs, Social Inequalities and Intervention Mechanisms	This topic focuses on the implementation of health programs, socially disadvantaged groups, multiple diseases, and community-based interventions.
	5	Effects of COVID-19 in Primary Health Care	The impact of COVID-19 on primary health care (family medicine, GPs), changes in practice, service delivery, and patient experiences are at the center of this topic.
	6	Effects of COVID-19 on Education and Medical Students	This topic covers the impact of the pandemic, especially on medical/health students, distance learning, internship placements, and learning outcomes.
CANADA	1	Impact of COVID-19 on Service Delivery in Family Medicine and Primary Care	This topic focuses on the impact of COVID-19 on primary health care, particularly family medicine practice, access to care, virtual consultations, and patient experiences. Particular attention has been paid to studies in regions such as Canada and Ontario.
	2	Clinical Risks, Chronic Diseases, and Health Professionals	This topic focuses on issues such as risks encountered in clinical practice, protection of health workers, coping with chronic diseases, and implementation of guidelines. Details on income level and socioeconomic variables are also covered under this heading.
	3	Clinical Findings, Training, and Comparative Analyses Post COVID-19	Under this heading, the focus is on the analysis of clinical impacts, case comparisons, and educational outcomes after COVID-19. The evaluation of acute and chronic complications and strategic responses was highlighted under this heading.
	4	Vaccination, Personal Protective Equipment, and Home-Based Impacts of COVID-19	The impacts of COVID-19 at home, access to vaccines, use of personal protective equipment, impacts on children, and practices in some regions were highlighted under this topic.
	5	Approaches, Challenges, and Mental Health in Health Service Delivery	This topic is related to the organization of health services, approaches to dealing with the pandemic, training of health workers, and policies related to mental health.
	6	Impact of COVID-19 on Families, Individuals, and Health Services	It covers the impact of the pandemic on families and individuals, interventions, surveys, and variables such as educational attainment. Evaluations in the Canadian (particularly Alberta) context have also been prominent in research.

### Open access to publications and funding sources

**Figure 7.**
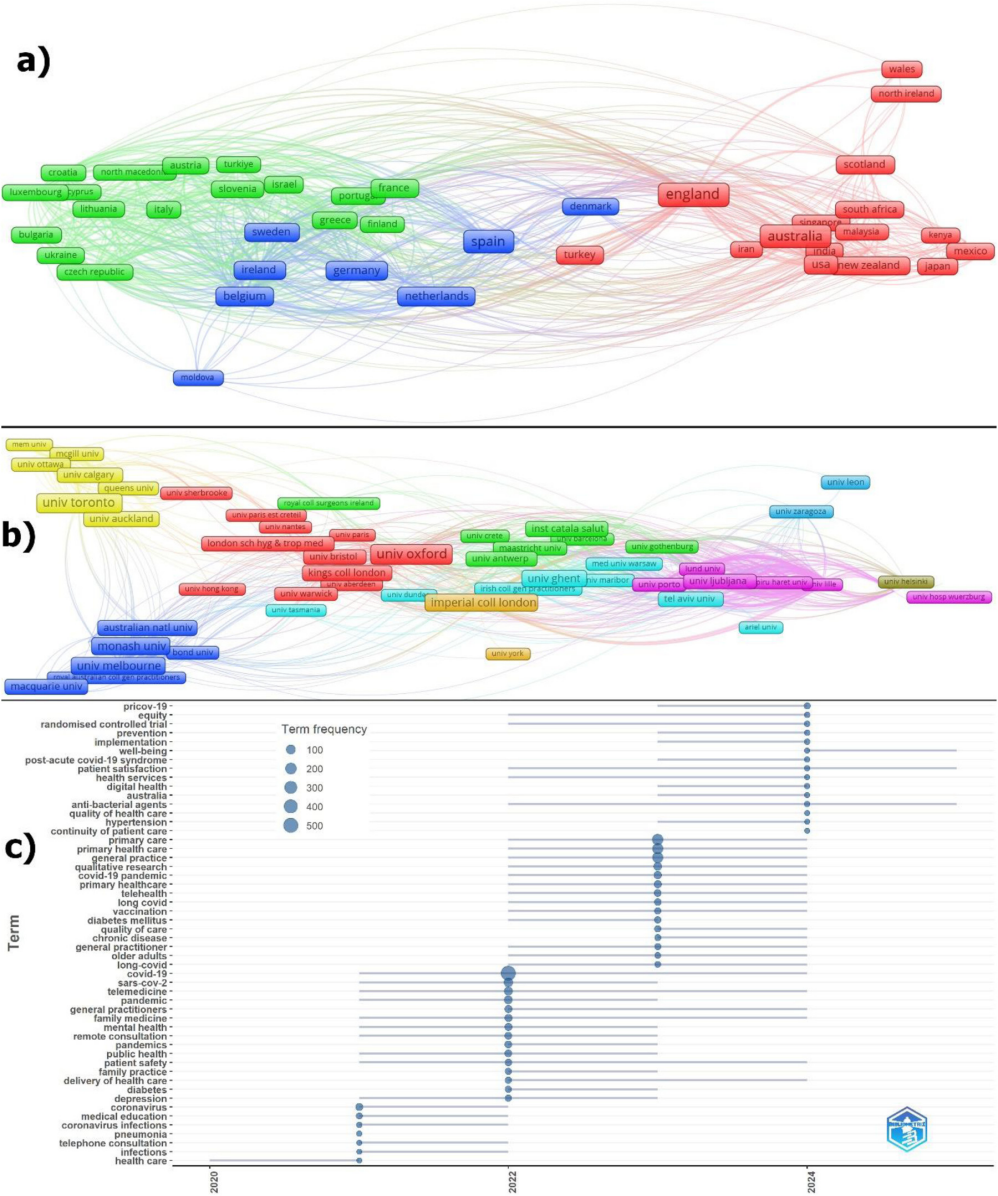
Co-Authorship country (a), co-authorship institutions (b), and author keywords trend topic (c) analyses for funded OECD countries’ documents.

Among the relevant articles, 88.40% (938 articles) were published as open access. The average number of citations of articles with open access is 9.14 and the h-index value is 41. In the articles without open access, the average number of citations per article is 4.25 and the h-index value is 11. In OECD countries, 547 articles (51.55%) lacked funding information, while this figure was 60 articles (50.42%) for Canadian research. Some articles were supported by more than one funding agency. Funding information, publication status, and economic data related to COVID-19 research in PHC services from OECD countries are presented in Table 9. Appendix 5 lists the institutions most actively supporting OECD researchers, while Appendix 6 details institutions supporting Canadian publications. Appendix 7 contains economic data specific to Canada. Funded articles for OECD countries were also analyzed by country, institution, and keywords, and the findings are presented in Figure 7.

**Table 9.** OECD countries foundation, publication, and some demographic and economics metrics (year: 2024).

Country	F1	F2	F3	F4	F5	F6	F7	F8	R1	R2	R3	R4	R5	R6	R7
Australia	1,855,856	1,051,004	5045	3040	20,511	10,496	161	85	26.929	14.036	4.259	1779.97	66,098.13	937.107	994.847
Austria	495,884	278,751	118	56	4982	2340	18	7	9.115	4.446	5.371	645.56	70,821.04	248.13	258.004
Belgium	689,401	395,942	575	259	6079	2815	50	8	11.734	5.233	5.744	793.825	67,649.72	306.647	335.483
Canada	2,371,713	1,412,856	6869	5538	23,473	9947	119	60	40.314	20.217	6.252	2471.99	61,317.93	1205.36	1224.27
Chile	236,100	114,858	75	36	3540	1911	3	0	20.086	n/a	9.032	620.883	30,910.65	75,521.41	79,497.30
Colombia	168,853	100,286	91	64	3249	1945	4	4	52.691	n/a	10.404	1059.70	20,111.33	556,773.25	597,985.63
Costa Rica	26,058	16,447	8	3	489	324	0	0	5.329	n/a	9.8	149.352	28,025.06	7820.40	9380.69
Czech Republic	362,224	158,572	41	13	2750	1003	8	2	11.087	5.021	2.6	564.19	50,889.17	3278.12	3456.30
Denmark	552,757	293,216	898	360	4467	1742	26	6	5.912	2.963	5	458.087	77,479.60	1460.26	1432.70
Estonia	52,126	20,836	35	12	567	203	3	0	1.348	0.704	7.078	63.856	47,383.44	15.969	17.273
Finland	434,294	237,308	388	191	3027	1142	10	2	5.633	2.639	7.448	346.967	61,600.32	151.085	158.401
France	2,598,399	1,726,217	515	261	14,510	7733	39	18	66.016	28.335	7.267	4009.50	60,734.90	1501.21	1631.88
Germany	3,432,228	2,025,981	871	344	23,114	9737	58	19	83.889	42.525	3.322	5715.26	68,128.88	1986.13	2057.21
Greece	366,716	250,893	194	110	4969	3428	20	6	10.455	4.362	9.255	434.833	41,592.73	108.625	110.418
Hungary	255,639	148,654	82	27	2367	1111	13	4	9.657	n/a	3.844	444.595	46,037.09	34,504.80	37,515.87
Iceland	30,503	14,090	43	27	301	113	0	0	0.389	0.222	3.793	28.172	72,491.84	1913.78	1967.89
Ireland	349,401	193,259	651	306	5356	2620	35	8	5.336	2.638	4.226	764.067	143,179.49	132.682	122.118
Israel	530,781	365,340	347	278	5400	3359	19	11	9.986	4.396	3.879	565.986	56,677.74	699.279	740.832
Italy	2,198,583	1,432,633	340	204	31,005	19,465	24	13	58.678	23.464	8	3286.91	56,016.32	995.051	1078.53
Japan	3,130,792	2,058,131	337	181	13,057	5202	25	12	124.04	67.519	2.3	6710.98	54,103.45	224,529.51	247,010.77
Korea	1,408,499	554,571	1101	937	11,057	4345	13	11	51.505	28.806	3.2	3056.74	59,348.56	560,492.48	581,256.22
Latvia	27,405	12,836	19	8	445	163	2	1	1.868	0.889	6.571	80.33	42,997.22	17.26	18.095
Lithuania	65,928	39,755	50	21	940	503	8	1	2.764	1.399	6.3	144.261	52,200.45	28.568	29.669
Luxembourg	27,819	11,464	14	4	556	220	7	2	0.67	0.518	5.755	97.726	145,825.71	37.754	39.393
Mexico	429,000	234,330	124	95	6155	3752	19	17	132.308	n/a	3.085	3423.59	25,875.82	8178.38	10,043.58
Netherlands	1,264,722	758,597	2393	1226	10,128	4324	61	21	17.761	9.59	4.1	1341.66	75,541.00	453.154	473.678
New Zealand	318,978	195,163	1002	509	3225	1774	43	23	5.242	2.948	4.891	288.484	55,034.48	162.453	177.073
Norway	420,254	223,950	834	401	3925	1733	28	9	5.541	2.875	3.8	470.17	84,850.74	3251.99	2397.05
Poland	855,213	523,631	1842	1692	8573	4864	37	21	37.554	n/a	2.897	1791.32	47,699.56	1605.81	1784.90
Portugal	390,235	165,785	231	139	5375	2253	17	10	10.295	5.152	6.475	482.851	46,903.03	118.164	118.309
Slovak Republic	121,732	58,102	14	8	1431	533	1	1	5.437	2.447	5.9	240.65	44,259.40	51.932	57.791
Slovenia	112,777	58,711	112	44	1354	591	18	8	2.116	1.102	3.776	113.604	53,698.49	29.226	31.077
Spain	1,801,758	971,177	2422	1927	21,556	11,241	170	122	47.994	20.931	11.341	2508.70	52,271.63	657.443	702.757
Sweden	907,146	499,154	1170	558	6775	2.338	31	10	10.91	5.198	8.077	736.745	67,529.82	3152.13	3193.96
Switzerland	940,931	531,135	375	168	8716	3687	20	7	8.871	5.311	2.34	820.699	92,519.28	264.961	261.623
Türkiye	879,405	651,195	316	255	16,092	13,629	43	35	87.237	n/a	10.135	3805.67	43,624.28	11,922.14	13,389.54
United Kingdom	3,968,638	2,459,325	8338	4906	46,677	21,301	321	113	68.434	32.809	4.638	3984.69	58,226.55	1090.17	1198.05
United States	15,260,765	9,285,068	22,559	17,904	125,303	62,295	64	35	336.692	161.889	3.849	27,966.55	83,062.62	8481.53	10,544.58

F1: article/review article count; F2: unfunded article/review article count; F3: PHC article/review article count; F4: unfunded PHC article/review article count; F5: COVID-19 article/review article count; F6: unfunded COVID-19 article/review article count; F7: COVID-19 article/review article count in PHC; F8: unfunded COVID-19 article/review article count in PHC; R1: population (2024) (millions); R2: employment (millions); R3: unemployment rate (percent of total labor force); R4: gross domestic product, current prices (billions) (purchasing power parity; international dollars); R5: gross domestic product per capita, current prices (purchasing power parity; international dollars); R6: general government revenue (billions); R7: general government total expenditure (billions).

It was observed that non-funded articles had an average citation rate of 8.16 and an h-index of 28. Funded articles were also found to benefit from a more extensive citation network. When country-specific research funds are evaluated, it is seen that English-speaking countries such as the UK, Australia, Canada, and the USA benefit more from such funds. As a result of the content analysis we conducted in our study, it was seen that there was no significant difference in subject matter between funded and non-funded research. However, it was observed that the articles that received funding had a higher citation average.

## Discussion

Canada ranks third in article productivity within PHC research among OECD countries, following the USA and the United Kingdom. In the combined domain of COVID-19 and PHC research, Canada also ranks forth, following the United Kingdom, Spain, and Australia. During the COVID-19 pandemic, Canada significantly increased its aid budgets, with donor governments emphasizing the need to distribute vaccines to developing countries, support hospital services, and provide assistance to the most vulnerable individuals in terms of income and livelihoods.^64^

As shown in Table 9 and Appendix 7, Canada maintains a robust economic structure and leads in PHC research. One of the key objectives of OECD is to assist governments in achieving prosperity through international cooperation and addressing poverty. The OECD also advises on how to understand emerging global developments and issues while providing actionable solutions.^65^ Analysis of co-authorship patterns reveals strong collaboration between New Zealand, USA, Australia, and the United Kingdom during the COVID-19 pandemic. This collaboration is promising for OECD countries, suggesting that these countries could lead international cooperation in future pandemics, enabling more effective international collaboration, with PHC services and higher education institutions playing a more significant role. Additionally, there is no existing literature examining the publication support and funding status of higher education institutions in the context of COVID-19 to this extent and detail.

Palmer and Small^12^ advocate for governments to invest in social safety net programs that target the most at-risk populations. Investments in job creation, education and training, paid work experience, early childhood care and education, housing, health, and mental health services are seen as vital for mitigating the pandemic's impacts. The OECD addresses both local and global policy issues, evaluating themes such as trade, social welfare, governance, development, taxation, transportation, science, technology, and innovation. OECD members spent around $12 billion on COVID-19-related activities, with initial efforts focused on health systems, humanitarian aid, and food security. The organization also supported strategies to address the economic and social repercussions of the pandemic.^64^ However, coordination among countries has been lacking and poor coordination undermined efforts to control the pandemic, as highlighted in previous research.^66^ PHC has been identified as a critical component in combating the virus.

According to data from The Global Health Security Tracking Site reports, nearly 80,000 projects were funded between 2014 and 2020 to support global health security efforts. However, a lack of funding for preparedness systems during the COVID-19 pandemic remains concerning.^67^ The likelihood of encountering future pandemics is high,^68^ making it essential for OECD countries to issue periodic project calls that promote international cooperation, particularly in areas like pandemics and climate crises. This proactive approach would better prepare the global community for future pandemics.

For OECD countries, a distributed information system integrating data from various platforms—such as a system capable of diagnosing COVID-19 from lung tomography images used during the pandemic—could play a significant role in reducing inequalities among the different healthcare systems of these nations.^69^ Deep learning, in particular, has shown extensive applicability in the healthcare sector.^70^ While machine learning enables computers to analyze comprehensive datasets, deep learning—a specific methodology within machine learning—excels in extracting meaningful patterns from such data.^69^ It is especially relevant for image processing, where lung tomography images were widely used for diagnosis during the COVID-19 pandemic.^71^

Deep learning systems demonstrated exceptional potential in providing solutions during the pandemic.^72^ However, the implementation and maintenance of such distributed systems inherently come with numerous infrastructural challenges.^73–75^ Nevertheless, it is crucial to recognize that the literature offers numerous studies addressing these challenges.^69,74,76^ Through the coordination of OECD countries, the development of AI-based conversational systems, such as ChatGPT, in anticipation of pandemics similar to COVID-19 could significantly simplify daily tasks for OECD citizens.^77^ Preparing such systems in advance and making them globally accessible would be of immense value. Such services will also contribute to a more equitable distribution of global health services. Specifically, these services could be assessed by experts in the field of PHC research and further refined to enhance preparedness for future pandemics. Among OECD countries, researchers from institutions such as the University of London, University of Oxford, University of Toronto, University of Melbourne, and Institut Català de la Salut, representing countries including the United Kingdom, Spain, Australia, Canada, Germany, and the United States, have been actively engaged in this area. Leveraging the expertise and knowledge of these researchers more effectively could further strengthen efforts in developing such systems.

Despite demonstrating significant productivity in PHC research during the COVID-19 period, Spain and Turkey could not find enough funding for their studies. In contrast, researchers from the United Kingdom, Australia, Canada, and Germany received substantial financial support during the same period. Notably, the United States, despite being one of the most prominent OECD countries, demonstrated relatively low productivity in PHC research, ranking below nations such as the United Kingdom, Spain, Australia, and Canada. Another interesting fact is that the UK and Canada are the two most important and productive countries in the field of PHC research on WoS in general. In addition, the University of Toronto and the University of London are the two major universities in the field that are prominent in the general PHC literature.

General practitioners play a vital role in the COVID-19 response, but many-faced significant challenges. For example, in Europe, the operations of general practitioners’ clinics were often uncertain, and reliable data were lacking.^78^ OECD countries could benefit from implementing comprehensive operational plans that coordinate efforts from primary care to other levels of healthcare during future pandemics. Additionally, many countries supported institutions with tax reductions, direct aid, credit guarantees, and other forms of financial assistance during the COVID-19 crisis.^10^ Research centers and higher education institutions also played a crucial role in combating the pandemic, as noted in several studies.^79–82^ However, our findings indicate a lack of sufficient support for open-access publications, particularly in the economic field, which hindered swift information dissemination. This trend was observed in many OECD countries, including Canada, which underscores the need for more robust support for open access to scientific data circulation during future crises.

Various topics such as burnout,^83,84^ workload,^83^ stress and anxiety,^85^ and health promotion^86,87^ have been extensively explored among healthcare professionals in OECD countries and in Canada. The OECD should continue supporting international collaboration to address healthcare system challenges during crises, provide funding for scientific activities, and sponsor regular health conferences in the field. Publishing periodic reports that evaluate the preparedness of all 38 OECD countries for challenges such as pandemics and climate change would also be highly beneficial.

Patients with chronic diseases such as cancer,^88–90^ diabetes,^91,92^ renal failure,^93^ and hypertension^94,95^ have emerged as significant topics within OECD countries. Special attention is also required for pregnant women and the elderly, given the prevalence of age-related diseases in these populations.^96^ Future pandemic preparedness in OECD countries must include measures that specifically address the needs of vulnerable groups. Furthermore, Tekerek et al.^97^ highlighted that OECD countries should adopt strategies suited to their available resources and chronic disease burdens.

During the COVID-19 pandemic, primary care experienced significant transformations.^98^ Research has highlighted important issues such as the roles of primary care providers,^99^ primary care clinics,^100^ telemedicine,^5,101^ and remote consultations.^100,102^ The relationship between patients and doctors changed significantly, with telehealth services playing a crucial role. Canada quickly adapted to the use of telemedicine during the pandemic.^103^ Duckett^104^ particularly noted that how the shift to telehealth transformed primary care. However, Diamond et al.^105^ pointed out some disadvantages, such as the lack of physical examinations and the challenges in maintaining patient–doctor rapport. Evaluating these services through scenario-based research could better prepare us for future pandemics. Another major challenge during the pandemic was the shortage of personal protective equipment.^106,107^ The OECD could benefit from proactive cooperation to manage the supply chain of critical items during future emergencies. Lavoie^108^ highlighted Canada's insufficient investment in basic sciences and the disconnect between laboratory research and the country's ability to produce vaccines and antiviral drugs. Similar issues are likely present in other OECD countries. Establishing supply chain management plans for health equipment across OECD countries, prioritizing internal production, and securing distribution channels during crises are critical areas that warrant research and planning.

Funding for COVID-19 research in PHC in Canada primarily came from national sources. However, Canada has significant institutions and researchers in PHC (see Table 2, Table 3, Appendix 1, Appendix 2), which could potentially make it a leader in PHC research within the OECD. Leveraging Canada's research infrastructure to secure international funding could further enhance its contributions. Instituting internal training programs within Canadian research institutions to better access international funds would be a strategic step forward. The limited output of some OECD countries in PHC research is concerning, given the critical role PHC services play in improving public health during pandemics. The OECD could encourage policies that promote greater scientific productivity in PHC research. Establishing a commission focused on PHC research would strengthen scientific collaboration among OECD countries and improve public health outcomes during future crises. For example, the tremendous transformation and importance of information technologies in our era, combined with the growing impact of artificial intelligence technologies in the healthcare sector, can lead to significant developments in primary healthcare services.^109,110^ Effective AI-supported information systems established within OECD countries can be designed to be highly beneficial not only for COVID-19 but also for global threats such as cancer.^111–114^

While Canada's productivity in COVID-19 research is strong compared to many OECD countries, its position in PHC research could be further enhanced. There are also issues with institutional affiliation inconsistencies in Canadian studies (see Table 3). For instance, researchers from the University of Toronto have listed affiliations such as Saint Michael's Hospital or Li Ka Shing Knowledge Institute, creating potential attribution problems. Canadian universities could benefit from implementing clearer corporate policies to standardize naming conventions and ensure consistent representation in global academic rankings. This is because research conducted by a researcher affiliated with the University of Toronto could be attributed to a different institution, potentially disadvantaging Canadian universities that consistently rank high in Times Higher Education and Academic Ranking of World Universities.

## Strengths and limitations

The primary reason for selecting the WoS bibliometric data source over Scopus is the inclusion of a specific category for the PHC research area within WoS. This highlights a significant limitation of the Scopus database, as it lacks a dedicated category for a research area as critical to public health as PHC. It is recommended that Scopus address this gap by incorporating PHC into its categories. Furthermore, funding information utilized in this study was derived from bibliometric data available in WoS. However, as demonstrated in Appendices 5 and 6, there are notable limitations in the quality of funding-related data, which may affect the robustness of the analyses. To enhance future funding studies, it would be beneficial to establish guidelines encouraging the detailed and accurate entry of funding data into journal systems indexed by WoS. By categorizing more detailed funding information (such as the funding country, institution, and amount) obtained from researchers using data sources such as WoS and Scopus, and by linking this funding information to institutions and countries, researchers will be able to conduct more detailed research in the future on scientific productivity and the funding institutions and countries. Another limitation of our study is the fact that the number of articles produced in the relevant field can be normalized according to the population or number of researchers in each country. As it is difficult to obtain this type of data, this process could not be carried out.

## Conclusions

In our study, COVID-19 research conducted by OECD countries and specifically Canada was analyzed in detail using bibliometric methods, including collaboration patterns, funding statuses, co-authorship, co-citation, thematic mapping, factorial analysis, topic dendrogram, and LDA machine learning technique. As known, the OECD is an organization established through the collaboration of 38 countries, including Canada. PHC is a research area that has actively and prominently engaged in combating COVID-19. Initially, experts in this research field faced numerous challenges. Therefore, within the OECD organization, this study represents the most comprehensive examination of how academia, particularly in the PHC research field, has responded and addressed various issues during such pandemics, and the extent to which publications in the WoS have received funding support.

Literature review reveals that this study represents one of the most comprehensive examinations of funding in the healthcare field. However, institutions such as Clarivate, Scopus, or PubMed could implement a more systematic approach to recording the funding agencies when scanning journals. At this point, obtaining information such as the country of the funding agency, the amount of funding, whether it is supported by multiple institutions, etc., from journal editors or actors involved in the publication process, would be highly valuable for evaluating funding information in research and analyzing scientific productivity to track financial resources.

In addressing infectious diseases, stronger collaboration and coordination mechanisms must be established. In this regard, among OECD countries, Canada stands in a leading position due to its economic strength and reputable educational institutions worldwide. Therefore, Canada can take the lead in assuming responsibility for establishing commissions and structures within OECD countries to be better prepared for future pandemics. Through such structures, guiding actions and policies can be implemented to strengthen the multilateral system for addressing global emergencies and ensuring sustainable development within OECD countries. This initiative could lead to better preparedness for future health crises. It has been observed that the inclusion of PHC experts in such commissions is crucial.

## Supplemental Material

sj-docx-1-dhj-10.1177_20552076251389341 - Supplemental material for Science mapping of COVID-19 contributions in primary health care by OECD countries: A machine learning approach Supplemental material, sj-docx-1-dhj-10.1177_20552076251389341 for Science mapping of COVID-19 contributions in primary health care by OECD countries: A machine learning approach by Muhammet Damar, Benita Hosseini, Andrew David Pinto, Omer Aydin and Umit Cali in DIGITAL HEALTH

## References

[1] Schmid BV Büntgen U Easterday WR , et al. Climate-driven introduction of the Black Death and successive plague reintroductions into Europe. Proc Natl Acad Sci USA 2015; 112: 3020–3025. 25713390 10.1073/pnas.1412887112 PMC4364181

[2] Aassve A Alfani G Gandolfi F , et al. Epidemics and trust: the case of the Spanish Flu. Health Econ 2021; 30: 840–857. 33554412 10.1002/hec.4218 PMC7986373

[3] Karlsson M Nilsson T Pichler S . The impact of the 1918 Spanish flu epidemic on economic performance in Sweden: an investigation into the consequences of an extraordinary mortality shock. J Health Econ 2014; 36: 1–9. 24721206 10.1016/j.jhealeco.2014.03.005

[4] Wong SY Zhang D Sit RW , et al. Impact of COVID-19 on loneliness, mental health, and health service utilisation: a prospective cohort study of older adults with multimorbidity in primary care. Br J Gen Pract 2020; 70: e817–e824. 10.3399/bjgp20X713021 PMC7523921 32988955

[5] Vidal-Alaball J Acosta-Roja R Hernández NP , et al. Telemedicine in the face of the COVID-19 pandemic. Aten PrIMaria 2020; 52: 418–422. 32402477 10.1016/j.aprim.2020.04.003 PMC7164871

[6] Chudik A Mohaddes K Pesaran MH , et al. A counterfactual economic analysis of Covid-19 using a threshold augmented multi-country model. J Int Money Finance 2021; 119: 102477. 34584324 10.1016/j.jimonfin.2021.102477 PMC8460619

[7] Chudik A Mohaddes K Raissi M . Covid-19 fiscal support and its effectiveness. Econ Lett 2021; 205: 109939. 36540861 10.1016/j.econlet.2021.109939 PMC9754820

[8] United Nations SDG. The Sustainable Development Goals Report: Special Edition. Towards a Rescue Plan for People and Planet. United Nations; 2023 [cited 2025 Jan 21]. Available from: https://unstats.un.org/sdgs/report/2023/The-Sustainable-Development-Goals-Report-2023.pdf

[9] Rathnayaka IW Khanam R Rahman MM . Fiscal support during the COVID-19 pandemic and its determinants: evidence for OECD countries. J Econ Policy Reform 2024; 27: 107–123.

[10] Benmelech E Tzur-Ilan N . The determinants of fiscal and monetary policies during the COVID-19 crisis. Nat Bureau Econ Res 2020; BER Working Paper Series: 27461.

[11] Wildman J . COVID-19 and income inequality in OECD countries. Eur J Health Econ 2021; 22: 455–462. 33590424 10.1007/s10198-021-01266-4 PMC7883879

[12] Palmer AN Small E . COVID-19 and disconnected youth: lessons and opportunities from OECD countries. Scand J Public Health 2021; 49: 779–789. 34030549 10.1177/14034948211017017

[13] Ansarinasab M Saghaian S . Outbound, inbound and domestic tourism in the post-COVID-19 era in OECD countries. Sustainability 2023; 15: 9412.

[14] Bulut T Top ME . Estimation of the size of the COVID-19 pandemic using the epidemiological wavelength model: results from OECD countries. Public Health 2023; 220: 172–178. 37329774 10.1016/j.puhe.2023.05.013 PMC10186978

[15] Sepulveda ER Brooker AS . Income inequality and COVID-19 mortality: age-stratified analysis of 22 OECD countries. SSM-Popul Health 2021; 16: 100904. 34584934 10.1016/j.ssmph.2021.100904 PMC8456048

[16] He Y Zhang Z . Energy and economic effects of the COVID-19 pandemic: evidence from OECD countries. Sustainability 2022; 14: 12043.

[17] Greene MW Roberts AP Frugé AD . Negative association between Mediterranean diet adherence and COVID-19 cases and related deaths in Spain and 23 OECD countries: an ecological study. Front Nutr 2021; 8: 591964. 33748170 10.3389/fnut.2021.591964 PMC7973012

[18] Leong C Howlett M Safaei M . Blame avoidance and credit-claiming dynamics in government policy communications: evidence from leadership tweets in four OECD countries during the 2020–2022 COVID-19 pandemic. Policy Soc 2023; 42: 564–585.

[19] Apergis E Apergis N . The impact of COVID-19 on economic growth: evidence from a Bayesian panel vector autoregressive (BPVAR) model. Appl Econ 2021; 53: 6739–6751.

[20] Lu L Zheng H Chen M , et al. Tackling carbon intensity with green finance in the covid-19-era: recommendations for OECD economies. Clim Change Econ 2022; 13: 2240014.

[21] Inoue Y . Relationship between high organ donation rates and COVID-19 vaccination coverage. Front Public Health 2022; 10: 855051. 35480588 10.3389/fpubh.2022.855051 PMC9038079

[22] Cooper ID . Bibliometrics basics. J Med Libr Assoc 2015; 103: 217. 26512226 10.3163/1536-5050.103.4.013 PMC4613387

[23] Verma S Gustafsson A . Investigating the emerging COVID-19 research trends in the field of business and management: a bibliometric analysis approach. J Bus Res 2020; 118: 253–261. 32834211 10.1016/j.jbusres.2020.06.057 PMC7330579

[24] Murillo J Villegas LM Ulloa-Murillo LM , et al. Recent trends on omics and bioinformatics approaches to study SARS-CoV-2: a bibliometric analysis and mini-review. Comput Biol Med 2021; 128: 104162. 33310371 10.1016/j.compbiomed.2020.104162 PMC7710474

[25] Zhao J Zhu J Huang C , et al. Uncovering the information immunology journals transmitted for COVID-19: a bibliometric and visualization analysis. Front Immunol 2022; 13: 1035151. 36405695 10.3389/fimmu.2022.1035151 PMC9670819

[26] Damar HT Bilik O Ozdagoglu G , et al. Scientometric overview of nursing research on pain management. Rev Lat Am Enfermagem 2018; 26: e3051. 10.1590/1518-8345.2581.3051 PMC6136548 30183876

[27] Yang K Qi H . The public health governance of the COVID-19 pandemic: a bibliometric analysis. Healthcare 2022; 10: 299–319. 35206913 10.3390/healthcare10020299 PMC8872432

[28] Soytas M Danacioglu YO Boz MY , et al. COVID-19 and urology: a bibliometric analysis of the literature. Int J Clin Pract 2021; 75: e14965. 10.1111/ijcp.14965 PMC8646722 34626151

[29] Xu SC Zhao XY Xing HP , et al. Cardiac involvement in COVID-19: a global bibliometric and visualized analysis. Front Cardiovasc Med 2022; 9: 955237. 35966543 10.3389/fcvm.2022.955237 PMC9365052

[30] Gupta BM Pal R Rohilla L , et al. Bibliometric analysis of diabetes research in relation to the COVID-19 pandemic. J Diabetol 2021; 12: 350–356.

[31] Rahim F Khakimova A Ebrahimi A , et al. Global scientific research on sars-cov-2 vaccines: a bibliometric analysis. Cell J (Yakhteh) 2021; 23: 523. 10.22074/cellj.2021.7794 PMC8588811 34837679

[32] Wattanapisit A Kotepui M Wattanapisit S , et al. Bibliometric analysis of literature on physical activity and COVID-19. Int J Environ Res Public Health 2022; 19: 7116. 35742364 10.3390/ijerph19127116 PMC9223140

[33] Lunardi CN Subrinho FL Freitas Barros MP , et al. Bibliometric analysis: nanotechnology and COVID-19. Curr Top Med Chem 2022; 22: 629–638. 35255795 10.2174/1568026622666220307125446

[34] Mohan S Thakur J Mohan C , et al. Journal of family medicine and primary care-A five year bibliometric analysis from 2016 to 2020. J Family Med Prim Care 2022; 11: 3613–3621. 36387676 10.4103/jfmpc.jfmpc_2086_21 PMC9648233

[35] Inoue M Fukahori H Matsubara M , et al. Latent dirichlet allocation topic modeling of free-text responses exploring the negative impact of the early COVID-19 pandemic on research in nursing. Jpn J Nurs Sci 2023; 20: e12520. 10.1111/jjns.12520 PMC9877805 36448530

[36] Han JW Kim JM Lee H . Topic modeling-based analysis of news keywords related to patients with diabetes during the COVID-19 pandemic. Healthcare 2023; 11: 957. 37046886 10.3390/healthcare11070957 PMC10094025

[37] Pasin O Pasin T . A bibliometric analysis of rheumatology and COVID-19 researches. Clin Rheumatol 2021; 40: 4735–4740. 34215906 10.1007/s10067-021-05844-y PMC8253236

[38] Lan X Yu H Cui L . Application of telemedicine in COVID-19: a bibliometric analysis. Front Public Health 2022; 10: 908756. 35719666 10.3389/fpubh.2022.908756 PMC9199898

[39] Guleid FH Oyando R Kabia E , et al. A bibliometric analysis of COVID-19 research in Africa. BMJ Glob Health 2021; 6: e005690. 10.1136/bmjgh-2021-005690 PMC8111873 33972261

[40] Gallegos M Cervigni M Consoli AJ , et al. COVID-19 in Latin America: a bibliometric analysis of scientific publications in health. Electron J Gen Med 2020; 17: em261.

[41] Kulkarni CA Wadhokar OC Naqvi WM . Changing trends in Covid-19 publication in India by bibliometrics analysis. J Family Med Prim Care 2022; 11: 7177–7179. 36993106 10.4103/jfmpc.jfmpc_1394_21 PMC10041301

[42] Blei DM Ng AY Jordan MI . Latent dirichlet allocation. J Mach Learn Res 2003; 3: 993–1022.

[43] Li X Lei L . A bibliometric analysis of topic modelling studies (2000–2017). J Inf Sci 2021; 47: 161–175.

[44] OECD. About the OECD [Internet]. 2024 [cited 2025 Jan 17]. Available from: https://www.oecd.org/about/

[45] Yasli G Damar M Özbiçakci Ş , et al. Primary care research on hypertension: a bibliometric analysis using machine-learning. Medicine 2024; 103: e40482. 10.1097/MD.0000000000040482 PMC11596423 39809211

[46] Pedregosa F Varoquaux G Gramfort A , et al. Scikit-learn: machine learning in Python. J Mach Learn Res 2011; 12: 2825–2830.

[47] Hardeniya N Perkins J Chopra D , et al. Natural language processing: python and NLTK. Birmingham and Mumbai: Packt Publishing Ltd, 2016.

[48] Srinivasa-Desikan B . Natural language processing and computational linguistics: A practical guide to text analysis with Python, Gensim, spaCy, and Keras. Packt Publishing Ltd, 2018.

[49] Yim A Chung C Yu A . Matplotlib for python developers: Effective techniques for data visualization with Python. Packt Publishing Ltd, 2018.

[50] Jin Y . Development of word cloud generator software based on python. Procedia Eng 2017; 174: 788–792.

[51] Negara ES Triadi D Andryani R . Topic modelling twitter data with latent Dirichlet allocation method. In: 2019 International Conference on Electrical Engineering and Computer Science (ICECOS), 2019 Oct 2–3, Batam, Indonesia, 2019 [cited 2025 Jan 17]. IEEE. Available from: https://ieeexplore.ieee.org/document/8984523

[52] Vayansky I Kumar SA . A review of topic modeling methods. Inf Syst 2020; 94: 101582.

[53] Deerwester S Dumais ST Furnas GW , et al. Indexing by latent semantic analysis. J Am Soc Inf Sci 1990; 41: 391–407.

[54] Hofmann T . Unsupervised learning by probabilistic latent semantic analysis. Mach Learn 2001; 42: 177–196.

[55] Gallagher RJ Reing K Kale D , et al. Anchored correlation explanation: topic modeling with minimal domain knowledge. Trans Assoc Comput Linguist 2017; 5: 529–542.

[56] Dai AM Storkey AJ . The supervised hierarchical Dirichlet process. IEEE Trans Pattern Anal Mach Intell 2014; 37: 243–255. 10.1109/TPAMI.2014.2315802 26353239

[57] Bogdanowicz A Guan C . Dynamic topic modeling of Twitter data during the COVID-19 pandemic. Plos one 2022; 17: e0268669. 10.1371/journal.pone.0268669 PMC9140268 35622866

[58] Wang Z Chen J Chen J , et al. Identifying interdisciplinary topics and their evolution based on BERTopic. Scientometrics 2023: 1–26.

[59] Wu X Nguyen T Luu AT . A survey on neural topic models: methods, applications, and challenges. Artif Intell Rev 2024; 57: 18.

[60] Bishop CM Nasrabadi NM . Pattern recognition and machine learning. New York: springer, 2006.

[61] Wallach H Mimno D McCallum A. Rethinking LDA: why priors matter. In NeurIPS (NIPS) 2009 - advances in neural information processing systems 22: 23rd annual conference on neural information processing systems 2009. Vancouver, British Columbia, Canada, 7–10 December 2009, pp.1–9.

[62] Shenton AK Hay-Gibson NV . Bradford's law and its relevance to researchers. Educ Inf 2009; 27: 217–230.

[63] Alvarado RU . Growth of literature on Bradford's law. Investigación Bibliotecológica: archivonomía. Bibliotecol Inf 2016; 30: 51–72.

[64] Marchant N . Foreign aid hit a record high last year: here’s what it means for the global pandemic recovery [Internet]. World Economic Forum; 2021 Apr [cited 2025 Jan 17]. Available from: https://www.weforum.org/agenda/2021/04/foreign-aid-2020-covid-19-oecd/

[65] Republic of Türkiye Ministry of Foreign Affairs. Türkiye’s relations with the Organization for Economic Co-operation and Development (OECD) [Internet]. [cited 2025 Jan 17]. Available from: https://www.mfa.gov.tr/oecd.en.mfa

[66] Sachs JD Karim SS Aknin L , et al. The Lancet Commission on lessons for the future from the COVID-19 pandemic. Lancet 2022; 400: 1224–1280. 36115368 10.1016/S0140-6736(22)01585-9 PMC9539542

[67] World Economic Forum. World Economic Forum [Internet]. 2023 [cited 2025 Jan 17]. Available from: https://www.weforum.org/

[68] Sarti TD Lazarini WS Fontenelle LF , et al. What is the role of primary health care in the COVID-19 pandemic? Epidemiol Serv Saúde 2020; 29: e2020166. 10.5123/s1679-49742020000200024 32348404

[69] Aminizadeh S Heidari A Dehghan M , et al. Opportunities and challenges of artificial intelligence and distributed systems to improve the quality of healthcare service. Artif Intell Med 2024; 149: 102779. 38462281 10.1016/j.artmed.2024.102779

[70] Heidari A Jafari Navimipour N Dag H , et al. Deepfake detection using deep learning methods: a systematic and comprehensive review. Wiley Interdiscip Rev Data Min Knowl Discov 2024; 14: e1520.

[71] Tenda ED Yulianti M Asaf MM , et al. The importance of chest CT scan in COVID-19. Acta Med Indones 2020; 52: 68. 32291374

[72] Heidari A Navimipour NJ Unal M , et al. The COVID-19 epidemic analysis and diagnosis using deep learning: a systematic literature review and future directions. Comput Biol Med 2022; 141: 105141. 34929464 10.1016/j.compbiomed.2021.105141 PMC8668784

[73] Baumgart DC . Digital advantage in the COVID-19 response: perspective from Canada’s largest integrated digitalized healthcare system. NPJ Digit Med 2020; 3: 114. 32923691 10.1038/s41746-020-00326-y PMC7459297

[74] Jabarulla MY Lee HN . A blockchain and artificial intelligence-based, patient-centric healthcare system for combating the COVID-19 pandemic: opportunities and applications. Healthcare 2021; 9: 1019. 34442156 10.3390/healthcare9081019 PMC8391524

[75] Filip R Gheorghita Puscaselu R Anchidin-Norocel L , et al. Global challenges to public health care systems during the COVID-19 pandemic: a review of pandemic measures and problems. J Pers Med 2022; 12: 1295. 36013244 10.3390/jpm12081295 PMC9409667

[76] Niakan Kalhori RS Bahaadinbeigy K Deldar K , et al. Digital health solutions to control the COVID-19 pandemic in countries with high disease prevalence: literature review. J Med Internet Res 2021; 23: e19473. 10.2196/19473 PMC7951053 33600344

[77] Heidari A Navimipour NJ Zeadally S , et al. Everything you wanted to know about ChatGPT: components, capabilities, applications, and opportunities. Internet Technol Lett 2024; 7: e530.

[78] Van Poel E Vanden Bussche P Klemenc-Ketis Z , et al. How did general practices organize care during the COVID-19 pandemic: the protocol of the cross-sectional PRICOV-19 study in 38 countries. BMC Prim Care 2022; 23: 1–1. 10.1186/s12875-021-01587-6 PMC8760114 35172744

[79] Chinnery PF Pearce JJ Kinsey AM , et al. How COVID-19 has changed medical research funding. Interf Focus 2021; 11: 20210025. 10.1098/rsfs.2021.0025 PMC8504879 34956595

[80] Chow JS Blight V Brown M , et al. Curious thing, an artificial intelligence (AI)-based conversational agent for COVID-19 patient management. Aust J Prim Health 2023; 29: 312–318. 36683166 10.1071/PY22045

[81] Turner E Johnson E Levin K , et al. Multi-disciplinary community respiratory team management of patients with chronic respiratory illness during the COVID-19 pandemic. NPJ Prim Care Respir Med 2022; 32: 26. 35963843 10.1038/s41533-022-00290-y PMC9375196

[82] Rawaf S Allen LN Stigler FL , et al. Lessons on the COVID-19 pandemic, for and by primary care professionals worldwide. Euro J Gen Pract 2020; 26: 129–133. 10.1080/13814788.2020.1820479 PMC7534357 32985278

[83] Baptista S Teixeira A Castro L , et al. Physician burnout in primary care during the COVID-19 pandemic: a cross-sectional study in Portugal. J Prim Care Community Health 2021; 12: 1–9. 10.1177/21501327211008437 PMC8044566 33840276

[84] Jefferson L Heathcote C Bloor K . General practitioner well-being during the COVID-19 pandemic: a qualitative interview study. BMJ Open 2023; 13: e061531. 10.1136/bmjopen-2022-061531 PMC9950583 36813497

[85] Ashcroft R Donnelly C Dancey M , et al. Primary care teams’ experiences of delivering mental health care during the COVID-19 pandemic: a qualitative study. BMC Fam Pract 2021; 22: 1–2. 34210284 10.1186/s12875-021-01496-8 PMC8248293

[86] Copp T Isautier JM Nickel B , et al. COVID-19 challenges faced by general practitioners in Australia: a survey study conducted in march 2021. Aust J Prim Health 2021; 27: 357–363. 34586061 10.1071/PY21165

[87] Prado LB Rodríguez PM . Análisis de los hábitos nutricionales entre los principales agentes sanitarios en promoción de la salud (médicos/as y enfermeros/as) de los servicios de urgencias en tiempos de la COVID-19. Med Fam Semergen 2022; 48: 154–162. 10.1016/j.semerg.2021.07.001 PMC8316062 34419342

[88] Archer S Calanzani N Honey S , et al. Impact of the COVID-19 pandemic on cancer assessment in primary care: a qualitative study of GP views. BJGP Open 2021; 5: 1–11. 10.3399/BJGPO.2021.0056 PMC8450883 34006530

[89] Ip A Black G Vindrola-Padros C , et al. Socioeconomic differences in help seeking for colorectal cancer symptoms during COVID-19: a UK-wide qualitative interview study. Br J Gen Pract 2022; 72: e472–e482. 10.3399/BJGP.2021.0644 PMC9256043 35636968

[90] Mora N Guiriguet C Cantenys R , et al. Cancer diagnosis in primary care after second pandemic year in Catalonia: a time-series analysis of primary care electronic health records covering about 5 million people. Fam Pract 2023; 40: 183–187. 35861148 10.1093/fampra/cmac083 PMC9384533

[91] Karatas S Yesim T Beysel S . Impact of lockdown COVID-19 on metabolic control in type 2 diabetes mellitus and healthy people. Prim Care Diabetes 2021; 15: 424–427. 33441263 10.1016/j.pcd.2021.01.003 PMC7834877

[92] Sujan MS Tasnim R Islam MS , et al. COVID-19-specific diabetes worries amongst diabetic patients: the role of social support and other co-variates. Prim Care Diabetes 2021; 15: 778–785. 34210639 10.1016/j.pcd.2021.06.009 PMC8226038

[93] Carratalá VP Górriz-Zambrano C Ariño CM , et al. COVID-19 y enfermedad cardiovascular y renal:¿ Dónde estamos?¿ Hacia dónde vamos? Med Fam Semergen 2020; 46: 78–87. 10.1016/j.semerg.2020.05.005 PMC7211659 32448633

[94] Schiffrin EL Flack JM Ito S , et al. Hypertension and COVID-19. Am J Hypertens 2020; 33: 373–374. 32251498 10.1093/ajh/hpaa057 PMC7184512

[95] Gallo G Calvez V Savoia C . Hypertension and COVID-19: current evidence and perspectives. High Blood Press Cardiovasc Prev 2022; 29: 115–123. 35184271 10.1007/s40292-022-00506-9 PMC8858218

[96] Tabish SA . COVID-19 pandemic: emerging perspectives and future trends. J Public Health Res 2020; 9: jphr-2020. 10.4081/jphr.2020.1786 PMC7282311 32550223

[97] Tekerek B Günaltay MM Ozler G , et al. Determinants of COVID-19 cases and deaths in OECD countries. J Public Health 2024; 32: 473–484. 10.1007/s10389-023-01820-9 PMC9880371 36721741

[98] Wanat M Hoste M Gobat N , et al. Transformation of primary care during the COVID-19 pandemic: experiences of healthcare professionals in eight European countries. Br J Gen Pract 2021; 71: e634–e642. 10.3399/BJGP.2020.1112 PMC8274627 33979303

[99] Donnelly C Ashcroft R Bobbette N , et al. Interprofessional primary care during COVID-19: a survey of the provider perspective. BMC Fam Pract 2021; 22: 1–2. 33535973 10.1186/s12875-020-01366-9 PMC7857097

[100] Parker RF Figures EL Paddison CA , et al. Inequalities in general practice remote consultations: a systematic review. BJGP Open 2021; 5: 1–7. 10.3399/BJGPO.2021.0040 PMC8278507 33712502

[101] Carrillo de Albornoz S Sia KL Harris A . The effectiveness of teleconsultations in primary care: systematic review. Fam Pract 2022; 39: 168–82. 34278421 10.1093/fampra/cmab077 PMC8344904

[102] Tuijt R Rait G Frost R , et al. Remote primary care consultations for people living with dementia during the COVID-19 pandemic: experiences of people living with dementia and their carers. Br J Gen Pract 2021; 71: e574–e582. 10.3399/BJGP.2020.1094 PMC8136581 33630749

[103] Jacobs P Bell NR Woudstra D . Can you afford to keep practising?: family medicine finances transformed by COVID-19 in Alberta. Can Fam Phys 2021; 67: e306–e311. 10.46747/cfp.6711e306 PMC8589146 34772724

[104] Duckett S . What should primary care look like after the COVID-19 pandemic? Aust J Prim Health 2020; 26: 207–211. 32454003 10.1071/PY20095

[105] Diamond L Kulasegaram K Murdoch S , et al. Impact of early waves of the COVID-19 pandemic on family medicine residency training: analysis of survey data. Can Fam Phys 2023; 69: 271–277. 10.46747/cfp.6904271 PMC10112711 37072215

[106] Pecchia L Piaggio D Maccaro A , et al. The inadequacy of regulatory frameworks in time of crisis and in low-resource settings: personal protective equipment and COVID-19. Health Technol 2020; 10: 1375–1383. 10.1007/s12553-020-00429-2 PMC7195610 32363133

[107] Garzotto F Ceresola E Panagiotakopoulou S , et al. COVID-19: ensuring our medical equipment can meet the challenge. Expert Rev Med Devices 2020; 17: 483–489. 32434400 10.1080/17434440.2020.1772757

[108] Lavoie M . A public health mission in Canada in response to the COVID-19 pandemic. Glob Health J 2022; 6: 231–236. 36593997 10.1016/j.glohj.2022.12.002 PMC9796350

[109] Damar M Pinto AD Erenay FS , et al. Impact of COVID-19 on primary health care research trends and suggestions for better services approaches via blockchain based applications: impact of COVID-19 on PHC & blockchain based applications. Blockchain Healthc Today 2025; 8: 400. 10.30953/bhty.v8.400 PMC12860442 41623341

[110] Damar M da Trindade TG Pinto AD . Scientific production in primary health care in Latin American and Caribbean countries (1980–2024): a web of science perspective. Aten PrIMaria 2025; 57: 103224. 40482345 10.1016/j.aprim.2025.103224 PMC12173108

[111] Farhoudian A Heidari A Shahhosseini R . A new era in colorectal cancer: artificial intelligence at the forefront. Comput Biol Med 2025; 196: 110926. 40818204 10.1016/j.compbiomed.2025.110926

[112] Toumaj S Heidari A Navimipour NJ . Leveraging explainable artificial intelligence for transparent and trustworthy cancer detection systems. Artif Intell Med 2025: 103243. 40839960 10.1016/j.artmed.2025.103243

[113] Damar M Damar HT Özbiçakci Ş , et al. Mapping intellectual structure and research hotspots of cancer studies in primary health care: a machine-learning-based analysis. Medicine 2025; 104: e41749. 10.1097/MD.0000000000041749 PMC11936571 40128045

[114] Wang H Toumaj S Heidari A , et al. Neurodegenerative disorders: a holistic study of the explainable artificial intelligence applications. Eng Appl Artif Intell 2025; 153: 110752.

