# Supplementary material for: Science mapping of COVID-19 contributions in primary health care by OECD countries: A machine learning approach
Source: Digit Health. 2025 Oct 27;11:20552076251389341. doi: 10.1177/20552076251389341 (PMC12576027; doi:10.1177/20552076251389341)
Supplement: sj-docx-1-dhj-10.1177_20552076251389341 - Supplemental material for Science mapping of COVID-19 contributions in primary health care by OECD countries: A machine learning approach [file sj-docx-1-dhj-10.1177_20552076251389341.docx]

**APPENDICES**

**Appendix 1.** Author List for OECD Countries (Minimum 6 Articles)

| **Rank** | **Authors** | **Country** | **N** | **%** | **Rank** | **Authors** | **Country** | **N** | **%** |
| --- | --- | --- | --- | --- | --- | --- | --- | --- | --- |
| 1 | Van Poel E | Belgium | 20 | 1.88 | 28 | Bakola M | Greece | 6 | 0.56 |
| 2 | Willems S | Belgium | 20 | 1.88 | 29 | Chlabicz S | Poland | 6 | 0.56 |
| 3 | Dykgraaf SH | Australia | 13 | 1.22 | 30 | Coenen S | Belgium | 6 | 0.56 |
| 4 | Kidd M | Australia | 12 | 1.13 | 31 | Colliers A | Belgium | 6 | 0.56 |
| 5 | Desborough J | Australia | 11 | 1.03 | 32 | Dambha-miller H | United Kingdom | 6 | 0.56 |
| 6 | Vinker S | Israel | 11 | 1.03 | 33 | De Lusignan S | United Kingdom | 6 | 0.56 |
| 7 | Butler CC | United Kingdom | 10 | 0.94 | 34 | Feder G | United Kingdom | 6 | 0.56 |
| 8 | Ares-blanco S | Spain | 9 | 0.84 | 35 | Fernández-villa T | Spain | 6 | 0.56 |
| 9 | Collins C | Ireland | 9 | 0.84 | 36 | Geersing GJ | Netherlands | 6 | 0.56 |
| 10 | Serrano-cumplido A | Spain | 9 | 0.84 | 37 | Goldacre B | United Kingdom | 6 | 0.56 |
| 11 | Brown JB | Canada | 8 | 0.75 | 38 | Hart N | United Kingdom | 6 | 0.56 |
| 12 | Goodyear-smith F | New Zealand | 8 | 0.75 | 39 | Inglesby P | United Kingdom | 6 | 0.56 |
| 13 | Goossens H | Belgium | 8 | 0.75 | 40 | Kiran T | Canada | 6 | 0.56 |
| 14 | Sturgiss E | Australia | 8 | 0.75 | 41 | Klemenc-ketis Z | Slovenia | 6 | 0.56 |
| 15 | Venekamp RP | Netherlands | 8 | 0.75 | 42 | Lionis C | Greece | 6 | 0.56 |
| 16 | Georgiou A | Australia | 7 | 0.66 | 43 | Majeed A | United Kingdom | 6 | 0.56 |
| 17 | Greenhalgh T | United Kingdom | 7 | 0.66 | 44 | Marshall EG | Canada | 6 | 0.56 |
| 18 | Hoffmann K |  | 7 | 0.66 | 45 | Mathews M | Canada | 6 | 0.56 |
| 19 | Mackenna B | United Kingdom | 7 | 0.66 | 46 | Meredith L | Canada | 6 | 0.56 |
| 20 | Magin P | Australia | 7 | 0.66 | 47 | Morton CE | United Kingdom | 6 | 0.56 |
| 21 | Mehrkar A | United Kingdom | 7 | 0.66 | 48 | Petek D | Slovenia | 6 | 0.56 |
| 22 | Nessler K | Poland | 7 | 0.66 | 49 | Seifert B | Czech Republic | 6 | 0.56 |
| 23 | Neves AL | United Kingdom | 7 | 0.66 | 50 | Sundvall PD | Sweden | 6 | 0.56 |
| 24 | Vargas MF | Spain | 7 | 0.66 | 51 | Thomas J | Australia | 6 | 0.56 |
| 25 | Vellinga A | Ireland | 7 | 0.66 | 52 | Torzsa P | Hungary | 6 | 0.56 |
| 26 | Astier-peña MP | Spain | 6 | 0.56 | 53 | Vaes B | Belgium | 6 | 0.56 |
| 27 | Bacon S | United Kingdom | 6 | 0.56 |  |  |  |  |  |

*N: Artice Count.*

**Appendix 2.** Author List for Canada (Minimum 4 Articles)

| **Rank** | **Researcher Profiles** | **Affiliations** | **Country** | **N** | **%** |
| --- | --- | --- | --- | --- | --- |
| 1 | Brown JB | Western University | Canada | 8 | 6.72 |
| 2 | Desborough J | Australian National University | Australia | 7 | 5.88 |
| 3 | Dykgraaf SH | Australian National University | Australia | 7 | 5.88 |
| 4 | Kidd M | University of New South Wales | Australia | 7 | 5.88 |
| 5 | Kiran T | University of Toronto | Canada | 6 | 5.04 |
| 6 | Marshall EG | Dalhousie University | Canada | 6 | 5.04 |
| 7 | Mathews M | Western University | Canada | 6 | 5.04 |
| 8 | Meredith L | Western University | Canada | 6 | 5.04 |
| 9 | Grad R | McGill University | Canada | 5 | 4.20 |
| 10 | Hedden L | Simon Fraser University | Canada | 5 | 4.20 |
| 11 | Leslie M | University of Calgary | Canada | 5 | 4.20 |
| 12 | Lukewich J | Memorial University Newfoundland | Canada | 5 | 4.20 |
| 13 | Ryan D | Western University | Canada | 5 | 4.20 |
| 14 | Spencer S | Simon Fraser University | Canada | 5 | 4.20 |
| 15 | Ashcroft R | University of Toronto | Canada | 4 | 3.36 |
| 16 | Donnelly C | Queen's University | Canada | 4 | 3.36 |
| 17 | Ebell MH | University of Georgia | USA | 4 | 3.36 |
| 18 | Eissa A | University of Toronto | Canada | 4 | 3.36 |
| 19 | Gill S | University of Toronto | Canada | 4 | 3.36 |
| 20 | Rayner J | University of Toronto | Canada | 4 | 3.36 |

** Affiliations information retrieved from WoS researcher profile, N: Artice Count.*

**Appendix 3.** Collaboration Countries List for OECD Countries (Minimum 2 Articles)

| **Rank** | **Countries** | **N** | **%** | **Rank** | **Countries** | **N** | **%** |
| --- | --- | --- | --- | --- | --- | --- | --- |
| 1 | England | 248 | 23.37 | 43 | Czech Republic | 8 | 0.75 |
| 2 | Spain | 170 | 16.02 | 44 | Ecuador | 8 | 0.75 |
| 3 | Australia | 161 | 15.17 | 45 | Lithuania | 8 | 0.75 |
| 4 | Canada | 119 | 11.21 | 46 | Romania | 8 | 0.75 |
| 5 | Usa | 64 | 6.03 | 47 | Luxembourg | 7 | 0.66 |
| 6 | Netherlands | 61 | 5.74 | 48 | Saudi Arabia | 7 | 0.66 |
| 7 | Germany | 58 | 5.46 | 49 | Bangladesh | 6 | 0.56 |
| 8 | Belgium | 50 | 4.71 | 50 | Bulgaria | 6 | 0.56 |
| 9 | New Zealand | 43 | 4.05 | 51 | Malaysia | 6 | 0.56 |
| 10 | France | 39 | 3.67 | 52 | Pakistan | 6 | 0.56 |
| 11 | Poland | 37 | 3.48 | 53 | Singapore | 6 | 0.56 |
| 12 | Scotland | 36 | 3.39 | 54 | Cyprus | 5 | 0.47 |
| 13 | Ireland | 35 | 3.29 | 55 | Kenya | 5 | 0.47 |
| 14 | Sweden | 31 | 2.92 | 56 | Moldova | 5 | 0.47 |
| 15 | Turkey | 29 | 2.73 | 57 | North Macedonia | 5 | 0.47 |
| 16 | Norway | 28 | 2.63 | 58 | Peru | 5 | 0.47 |
| 17 | Denmark | 26 | 2.45 | 59 | Philippines | 5 | 0.47 |
| 18 | Japan | 25 | 2.35 | 60 | Belarus | 4 | 0.37 |
| 19 | Italy | 24 | 2.26 | 61 | Colombia | 4 | 0.37 |
| 20 | Wales | 23 | 2.16 | 62 | Egypt | 4 | 0.37 |
| 21 | India | 21 | 1.97 | 63 | Georgia | 4 | 0.37 |
| 22 | Greece | 20 | 1.88 | 64 | Indonesia | 4 | 0.37 |
| 23 | Switzerland | 20 | 1.88 | 65 | Lebanon | 4 | 0.37 |
| 24 | Israel | 19 | 1.79 | 66 | Nepal | 4 | 0.37 |
| 25 | Mexico | 19 | 1.79 | 67 | Serbia | 4 | 0.37 |
| 26 | Austria | 18 | 1.69 | 68 | Argentina | 3 | 0.28 |
| 27 | Peoples R China | 18 | 1.69 | 69 | Bosnia Herceg | 3 | 0.28 |
| 28 | Slovenia | 18 | 1.69 | 70 | Chile | 3 | 0.28 |
| 29 | South Africa | 18 | 1.69 | 71 | Estonia | 3 | 0.28 |
| 30 | Portugal | 17 | 1.60 | 72 | Sri Lanka | 3 | 0.28 |
| 31 | North Ireland | 14 | 1.32 | 73 | Brazil | 2 | 0.18 |
| 32 | Turkiye | 14 | 1.32 | 74 | Cuba | 2 | 0.18 |
| 33 | Hungary | 13 | 1.22 | 75 | Ghana | 2 | 0.18 |
| 34 | South Korea | 13 | 1.22 | 76 | Iraq | 2 | 0.18 |
| 35 | Finland | 10 | 0.94 | 77 | Latvia | 2 | 0.18 |
| 36 | Iran | 9 | 0.84 | 78 | Nigeria | 2 | 0.18 |
| 37 | Ukraine | 9 | 0.84 | 79 | Qatar | 2 | 0.18 |
| 38 | Croatia | 8 | 0.75 | 80 | Syria | 2 | 0.18 |

*N: Artice Count.*

**Appendix 4.** Collaboration Countries List for Canada

| **Rank** | **Countries** | **N** | **%** |
| --- | --- | --- | --- |
| 1 | Canada | 119 | 100.00 |
| 2 | Usa | 13 | 10.92 |
| 3 | Australia | 11 | 9.24 |
| 4 | Switzerland | 5 | 4.20 |
| 5 | England | 4 | 3.36 |
| 6 | India | 4 | 3.36 |
| 7 | New Zealand | 4 | 3.36 |
| 8 | France | 3 | 2.52 |
| 9 | Greece | 3 | 2.52 |
| 10 | Iran | 3 | 2.52 |
| 11 | Peoples R China | 3 | 2.52 |
| 12 | Lebanon | 2 | 1.68 |
| 13 | Pakistan | 2 | 1.68 |
| 14 | Scotland | 2 | 1.68 |
| 15 | South Africa | 2 | 1.68 |
| 16 | Argentina | 1 | 0.84 |
| 17 | Ecuador | 1 | 0.84 |
| 18 | Egypt | 1 | 0.84 |
| 19 | Germany | 1 | 0.84 |
| 20 | Ireland | 1 | 0.84 |
| 21 | Kenya | 1 | 0.84 |
| 22 | Malaysia | 1 | 0.84 |
| 23 | Netherlands | 1 | 0.84 |
| 24 | North Ireland | 1 | 0.84 |
| 25 | Peru | 1 | 0.84 |
| 26 | Philippines | 1 | 0.84 |
| 27 | Poland | 1 | 0.84 |
| 28 | Portugal | 1 | 0.84 |
| 29 | Qatar | 1 | 0.84 |
| 30 | Saudi Arabia | 1 | 0.84 |
| 31 | Sweden | 1 | 0.84 |
| 32 | Tanzania | 1 | 0.84 |
| 33 | Zambia | 1 | 0.84 |

**Appendix 5.** Funding Organizations List for OECD Countries (Minimum 2 Articles)

| **Rank** | **Funding Agencies** | **N** | **%** | **Rank** | **Funding Agencies** | **N** | **%** |
| --- | --- | --- | --- | --- | --- | --- | --- |
| 1 | UK Research Innovation | 40 | 3.77 | 22 | Spanish Government | 5 | 0.47 |
| 2 | National Institutes Of Health Research | 39 | 3.67 | 23 | Department Of Industry Innovation And Science | 4 | 0.37 |
| 3 | Medical Research Council UK | 23 | 2.16 | 24 | Fonds De Recherche Du Quebec | 4 | 0.37 |
| 4 | European Union | 17 | 1.60 | 25 | Fonds De Recherche Du Quebec Sante Frqs | 4 | 0.37 |
| 5 | Wellcome Trust | 17 | 1.60 | 26 | German Research Foundation DFG | 4 | 0.37 |
| 6 | Australian Government | 16 | 1.50 | 27 | Health Research Council Of New Zealand | 4 | 0.37 |
| 7 | National Health Medical Research Council Of Australia | 16 | 1.50 | 28 | Horizon 2020 | 4 | 0.37 |
| 8 | Netherlands Organization For Health Research And Development | 16 | 1.50 | 29 | Instituto De Salud Carlos III | 4 | 0.37 |
| 9 | Canadian Institutes Of Health Research Cihr | 13 | 1.22 | 30 | Acev Foundation | 3 | 0.28 |
| 10 | Projekt Deal | 12 | 1.13 | 31 | British Heart Foundation | 3 | 0.28 |
| 11 | European General Practice Research Network Egprn | 11 | 1.03 | 32 | Cooperative Research Centres Programme | 3 | 0.28 |
| 12 | Health Foundation | 8 | 0.75 | 33 | Engineering Physical Sciences Research Council | 3 | 0.28 |
| 13 | Ministry Of Education Culture Sports Science And Technology Japan Mext | 8 | 0.75 | 34 | European Research Council | 3 | 0.28 |
| 14 | Cancer Research UK | 7 | 0.66 | 35 | Federal Ministry Of Education Research Bmbf | 3 | 0.28 |
| 15 | Japan Society For The Promotion Of Science | 7 | 0.66 | 36 | Michael Smith Foundation For Health Research | 3 | 0.28 |
| 16 | Economic Social Research Council Esrc | 6 | 0.56 | 37 | National Institute For Health And Care Research | 3 | 0.28 |
| 17 | Grants In Aid For Scientific Research Kakenhi | 6 | 0.56 | 38 | Pfizer | 3 | 0.28 |
| 18 | Australian Department Of Health | 5 | 0.47 | 39 | Public Health England Phe | 3 | 0.28 |
| 19 | European Commission Joint Research Centre | 5 | 0.47 | 40 | Royal Melbourne Hospital Foundation | 3 | 0.28 |
| 20 | Health Research Board Ireland | 5 | 0.47 | 41 | Sanofi | 3 | 0.28 |
| 21 | Royal Australian College Of General Practitioners | 5 | 0.47 | - | - | - | 0.- |

*N: Artice Count.*

**Appendix 6.** Funding Organizations List for Canada

| **Rank** | **Funding Agencies** | **N** | **%** | **Rank** | **Funding Agencies** | **N** | **%** |
| --- | --- | --- | --- | --- | --- | --- | --- |
| 1 | Canadian Institutes Of Health Research Cihr | 13 | 10.92 | 51 | Inspire Primary Health Care Research Program | 1 | 0.84 |
| 2 | Fonds De Recherche Du Quebec Frq | 4 | 3.36 | 52 | Inspire Primary Health Care Research Program Through The Health Systems Research Program Of The Ontario Ministry Of Health And Long Term Care | 1 | 0.84 |
| 3 | Fonds De Recherche Du Quebec Sante Frqs | 4 | 3.36 | 53 | Institut Canadien D Information Sur La Sant | 1 | 0.84 |
| 4 | Michael Smith Foundation For Health Research | 3 | 2.52 | 54 | Interior Universities Research Coalition | 1 | 0.84 |
| 5 | Foundation Of Advancing Family Medicine | 2 | 1.68 | 55 | KPMG | 1 | 0.84 |
| 6 | Ices Ontario Ministry Of Health | 2 | 1.68 | 56 | Lawson Health Research Institute | 1 | 0.84 |
| 7 | Inspire Phc Applied Health Research Question | 2 | 1.68 | 57 | Li Ka Shing Knowledge Institute St Michael S Hospital | 1 | 0.84 |
| 8 | International Grenfell Association | 2 | 1.68 | 58 | Manitoba Covid 19 Rapid Response Research Grant Research Manitoba | 1 | 0.84 |
| 9 | Ministry Of Long Term Care | 2 | 1.68 | 59 | Manitoba Medical Services Foundation Operating Grant | 1 | 0.84 |
| 10 | Ministry Of Long Term Care | 2 | 1.68 | 60 | Mcgill Pbrn | 1 | 0.84 |
| 11 | Mitacs | 2 | 1.68 | 61 | Mclaughlin Foundation Professorship In Population And Public Health | 1 | 0.84 |
| 12 | Natural Sciences And Engineering Research Council Of Canada | 2 | 1.68 | 62 | Member Interest Group Mig Grant By The College Of Family Physicians Of Canada Cfpc | 1 | 0.84 |
| 13 | Ontario Ministere De La Sante Et Des Soins De Longue Duree | 2 | 1.68 | 63 | Ministere De La Sante Et Des Services Sociaux Du Quebec | 1 | 0.84 |
| 14 | Academic Medical Organization Of Southwestern Ontario | 1 | 0.84 | 64 | Mitacs Globalink Research Internship Award | 1 | 0.84 |
| 15 | Alberta Innovates | 1 | 0.84 | 65 | Montreal Children S Hospital Foundation | 1 | 0.84 |
| 16 | Anglo America Plc South Africa | 1 | 0.84 | 66 | Montreal General Hospital Foundation | 1 | 0.84 |
| 17 | Bill And Melinda Gates Foundation Through The Primary Health Care Research Consortium | 1 | 0.84 | 67 | National Health Medical Research Council Nhmrc Of Australia | 1 | 0.84 |
| 18 | Bill Melinda Gates Foundation | 1 | 0.84 | 68 | National Mental Health Commission | 1 | 0.84 |
| 19 | Blinded Institution Foundation | 1 | 0.84 | 69 | New Brunswick Innovation Fund | 1 | 0.84 |
| 20 | Canada Research Chair Tier 2 In Pediatric Brain Development And Rehabilitation | 1 | 0.84 | 70 | Ontario Ministry Of Health And Long Term Care Inspire Phc Applied Health Research Question Ahrq | 1 | 0.84 |
| 21 | Canada Research Chairs | 1 | 0.84 | 71 | Ontario Ministry Of Health Omoh | 1 | 0.84 |
| 22 | Canadian Consortium On Neurodegeneration In Aging | 1 | 0.84 | 72 | Opal Health Informatics Group At The Research Institute Of The Blinded Institution | 1 | 0.84 |
| 23 | Canadian Frailty Network Interdisciplinary Fellowship | 1 | 0.84 | 73 | Physician Assistant Mcmaster Family Practice | 1 | 0.84 |
| 24 | Cedars Cancer Foundation | 1 | 0.84 | 74 | Physicians Services Incorporated Foundation And As The Associate Director For Clinical Research At University Of Toronto Practice Based Research Network Utopian | 1 | 0.84 |
| 25 | Center For Interdisciplinary Research In Rehabilitation And Social Integration Cirris | 1 | 0.84 | 75 | Primary Care Clinics Across Hamilton | 1 | 0.84 |
| 26 | Centre De Recherche Du Centre Hospitalier De L Universite De Montreal | 1 | 0.84 | 76 | Public Health Agency Of Canada And The Medical Psychiatry Alliance | 1 | 0.84 |
| 27 | Centre For Collaboration Motivation And Innovation Ccmi | 1 | 0.84 | 77 | Public Health Agency Of Canada Chair In Applied Public Health Research | 1 | 0.84 |
| 28 | Cervo Brain Research Center | 1 | 0.84 | 78 | Public Safety Canada | 1 | 0.84 |
| 29 | Champlain Econsult Basetm | 1 | 0.84 | 79 | Quadruple Aim And Equity | 1 | 0.84 |
| 30 | Ciusss De La Capitale Nationale Vitam Research Center On Sustainable Health | 1 | 0.84 | 80 | Quebec Integrated University Health And Social Services Center | 1 | 0.84 |
| 31 | Clinical Programs And Innovation Central | 1 | 0.84 | 81 | Quebec Population Health Research Network | 1 | 0.84 |
| 32 | Cma Foundation Cmaf | 1 | 0.84 | 82 | Reseau 1 | 1 | 0.84 |
| 33 | Cma Foundation Cmaf For The Covid 19 Pandemic Response And Impact Grant Co Rig Program Phase I | 1 | 0.84 | 83 | Richard B Splane Applied Social Policy And Social Innovation Fund From The Factor Inwentash Faculty Of Social Work At The University Of Toronto | 1 | 0.84 |
| 34 | Cmdp Physician Research Fund Of The Jewish General Hospital Montreal | 1 | 0.84 | 84 | Schulich School Of Medicine And Dentistry | 1 | 0.84 |
| 35 | Commonwealth Fund | 1 | 0.84 | 85 | St Michael S Hospital Foundation | 1 | 0.84 |
| 36 | Commonwealth Fund For Mylaine Breton S Harkness Fellowship In Health Care Policy And Practice | 1 | 0.84 | 86 | Suffolk University | 1 | 0.84 |
| 37 | Covid 19 Pandemic Response And Impact Grant Co Rig Phase 1 Program | 1 | 0.84 | 87 | The Authors Wish To Acknowledge The Health Care Providers Who Took Time During Their Busy Schedules To Speak With Us To The Older Adult Participants Thank You For Sharing Your Experiences With Us | 1 | 0.84 |
| 38 | David Braley Chair In Family Medicine | 1 | 0.84 | 88 | The Authors Wish To Thank Jean Johnstone Qian Yang Ria De Gorter Dr Anne Cranney And Dr Giuseppe Ficara For Their Support And Contributions | 1 | 0.84 |
| 39 | Department Of Family And Community Medicine At The University Of Toronto | 1 | 0.84 | 89 | The Authors Would Like To Acknowledge The Prevention Practitioners Who Worked On Better Wise A Browne C Cole L Coughlin S French D Gallant J Gheseger V Hans M Lajeunesse M Larsen K Lilly S Moshiri M Rathnavalu S Sohanpal T Stain | 1 | 0.84 |
| 40 | Dept Of Family And Community Medicine Faculty Of Medicine At University Of Toronto And St Michael S Hospital | 1 | 0.84 | 90 | The Blinded Institution Interdisciplinary Initiative In Infection And Immunity M I 4 Emergency Covid 19 Research Funding | 1 | 0.84 |
| 41 | Factor Inwentash Faculty Of Social Work University Of Toronto | 1 | 0.84 | 91 | Tier 1 Canada Research Chair In Population Health Intervention Research | 1 | 0.84 |
| 42 | Faculty Of Medicine Memorial University Of Newfoundland | 1 | 0.84 | 92 | Tier 2 Canada Research Chair In Urban Health Equity Among Young People | 1 | 0.84 |
| 43 | Fonds De Recherche Du Quebec Nature Et Technologies Frqnt | 1 | 0.84 | 93 | Ubc Children S And Women S Research Ethics Board | 1 | 0.84 |
| 44 | Foundation For Advancing Family Medicine | 1 | 0.84 | 94 | Umea University | 1 | 0.84 |
| 45 | French Network Of University Hospitals Hugo Hopitaux Universitaires Du Grand Ouest | 1 | 0.84 | 95 | University Center For Research On Youth And Families Crujef | 1 | 0.84 |
| 46 | Harvard Medical School | 1 | 0.84 | 96 | University Of Toronto | 1 | 0.84 |
| 47 | Health Centre Of The Provincial Health Services Authority Of British Columbia | 1 | 0.84 | 97 | Vancouver Coastal Health Research Institute Vchri | 1 | 0.84 |
| 48 | Health Resources And Services Administration Hrsa Of The U S Department Of Health And Human Services | 1 | 0.84 | 98 | Western University | 1 | 0.84 |
| 49 | Health Systems Research Program Of The Ontario Ministry Of Health | 1 | 0.84 | 99 | Western University Catalyst Grant Surviving Pandemics | 1 | 0.84 |
| 50 | Ices Ontario Ministry Of Health | 1 | 0.84 | 100 | Women S Health Research Institute Of The British Columbia Women S Hospital | 1 | 0.84 |

*N: Artice Count.*

**Appendix 7.** Canadian Government Some Economic Statistics During The COVID-19

| **Subject Descriptor** | **Units** | **Scale** | **2019** | **2020** | **2021** | **2022** | **2023** | **2024** | **2025** | **Estimates Start After** |
| --- | --- | --- | --- | --- | --- | --- | --- | --- | --- | --- |
| Gross domestic product, constant prices | National currency | Billions | 2,109.99 | 2,002.92 | 2,103.31 | 2,175.62 | 2,203.73 | 2,239.16 | 2,292.40 | 2022 |
| Gross domestic product, current prices | National currency | Billions | 2,313.56 | 2,209.68 | 2,509.62 | 2,782.65 | 2,842.79 | 2,966.26 | 3,095.43 | 2022 |
| Gross domestic product, current prices | U.S. dollars | Billions | 1,743.73 | 1,647.60 | 2,001.49 | 2,137.94 | 2,117.81 | 2,238.57 | 2,364.55 | 2022 |
| Gross domestic product, current prices | Purchasing power parity; international dollars | Billions | 1,939.59 | 1,865.20 | 2,046.66 | 2,265.32 | 2,378.97 | 2,471.99 | 2,581.77 | 2022 |
| Gross domestic product, deflator | Index |  | 109.648 | 110.323 | 119.318 | 127.901 | 128.999 | 132.472 | 135.03 | 2022 |
| Gross domestic product per capita, constant prices | National currency | Units | 56,206.69 | 52,739.93 | 55,052.86 | 56,006.44 | 55,407.28 | 55,542.71 | 56,132.64 | 2022 |
| Gross domestic product per capita, constant prices | Purchasing power parity; 2017 international dollar | Units | 49,565.49 | 46,508.36 | 48,547.99 | 49,388.90 | 48,860.54 | 48,979.97 | 49,500.20 | 2022 |
| Gross domestic product per capita, current prices | National currency | Units | 61,629.50 | 58,184.18 | 65,687.87 | 71,633.05 | 71,474.81 | 73,578.60 | 75,796.01 | 2022 |
| Gross domestic product per capita, current prices | U.S. dollars | Units | 46,449.96 | 43,383.71 | 52,387.81 | 55,036.52 | 53,246.98 | 55,527.99 | 57,899.39 | 2022 |
| Gross domestic product per capita, current prices | Purchasing power parity; international dollars | Units | 51,667.53 | 49,113.43 | 53,570.17 | 58,315.66 | 59,813.41 | 61,317.93 | 63,218.29 | 2022 |
| Gross domestic product based on purchasing-power-parity (PPP) share of world total | Percent |  | 1.428 | 1.398 | 1.381 | 1.383 | 1.361 | 1.344 | 1.334 | 2022 |
| Implied PPP conversion rate | National currency per current international dollar |  | 1.193 | 1.185 | 1.226 | 1.228 | 1.195 | 1.2 | 1.199 | 2022 |
| General government revenue | National currency | Billions | 938.667 | 923.632 | 1,040.86 | 1,130.29 | 1,156.06 | 1,205.36 | 1,258.22 | 2022 |
| General government total expenditure | National currency | Billions | 939.067 | 1,164.72 | 1,150.81 | 1,152.93 | 1,176.74 | 1,224.27 | 1,274.04 | 2022 |
| General government net lending/borrowing | National currency | Billions | -0.4 | -241.085 | -109.956 | -22.642 | -20.68 | -18.914 | -15.817 | 2022 |
| General government structural balance | National currency | Billions | -5.367 | -185.719 | -84.556 | -37.486 | -22.655 | -11.861 | -16.193 | 2022 |
| General government primary net lending/borrowing | National currency | Billions | 2.397 | -231.337 | -124.635 | -34.804 | -27.564 | -20.885 | -15.783 | 2022 |
| General government net debt | National currency | Billions | 196.033 | 347.515 | 385.925 | 395.242 | 415.922 | 431.869 | 447.686 | 2022 |
| General government gross debt | National currency | Billions | 2,087.07 | 2,626.70 | 2,887.92 | 2,987.87 | 3,024.15 | 3,065.44 | 3,114.74 | 2022 |
| Gross domestic product corresponding to fiscal year, current prices | National currency | Billions | 2,313.56 | 2,209.68 | 2,509.62 | 2,782.65 | 2,842.79 | 2,966.26 | 3,095.43 | 2022 |
| Unemployment rate | Percent of total labor force |  | 5.7 | 9.725 | 7.508 | 5.275 | 5.527 | 6.252 | 6.023 | 2022 |
| Employment | Persons | Millions | 19.114 | 18.047 | 18.95 | 19.7 | 20.088 | 20.217 | n/a | 2022 |
| Population | Persons | Millions | 37.54 | 37.977 | 38.205 | 38.846 | 39.773 | 40.314 | 40.839 | 2022 |

**Sources:** https://www.imf.org/en/Publications/WEO/weo-database/2024/October/select-country-group
